# Explainable machine learning for perioperative surgical site infection risk enrichment after operative treatment of closed pilon fractures: a multicenter retrospective study with external validation

**DOI:** 10.3389/fsurg.2026.1850385

**Published:** 2026-07-06

**Authors:** Zhigang Deng, Feifei Zhao, Yanci Zhang, Tao Zhang, Xuebin Zhang, Yang Luo

**Affiliations:** 1 Trauma Orthopaedics Ward 2, Third Hospital of Shijiazhuang, Shijiazhuang, China; 2 Department of Orthopaedic Surgery, The Third Hospital of Hebei Medical University, Shijiazhuang, China; 3 Trauma Center, The First Hospital of Hebei Medical University, Shijiazhuang, China

**Keywords:** calibration, closed pilon fractures, external validation, machine learning, risk stratification, surgical site infection, targeted surveillance

## Abstract

**Background:**

Surgical site infection (SSI) remains a major complication after operative treatment of closed pilon fractures. Prediction tools may help identify patients who warrant closer perioperative wound surveillance, but externally validated and clinically interpretable models remain limited. We aimed to develop and externally validate explainable machine-learning models for perioperative SSI risk enrichment after operative treatment of closed pilon fractures.

**Methods:**

This retrospective multicenter study included 1,876 patients treated at the internal center between January 2020 and December 2024 and 359 patients from an independent external center treated between August 2024 and September 2025. The internal dataset was stratified into a training set and an internal test set at a 7:3 ratio. Candidate predictors included demographic, injury-related, perioperative, laboratory, and inflammatory-metabolic variables. LASSO regression was used for feature selection, followed by logistic regression, decision tree, random forest (RF), XGBoost, and naïve Bayes models. Thresholds were optimized using out-of-fold cross-validation predictions. Model performance was evaluated by ROC-AUC, PR-AUC, sensitivity, specificity, precision, F1 score, balanced accuracy, calibration indices, Brier score, bootstrap 95% confidence intervals, and decision-curve analysis. A preoperative-only RF sensitivity model was also constructed.

**Results:**

The internal cohort included 74 SSI events (3.9%), and the external cohort included 11 SSI events (3.1%). LASSO retained 16 predictors. The RF model showed the highest overall discrimination, with ROC-AUCs of 0.899 (95% CI, 0.831–0.953) in the internal test set and 0.902 (95% CI, 0.707–0.991) in the external cohort. Sensitivity was 0.294 (95% CI, 0.077–0.533) internally and 0.636 (95% CI, 0.333–0.909) externally. RF specificity was high, at 0.987 (95% CI, 0.976–0.996) and 0.974 (95% CI, 0.957–0.989), respectively. RF Brier scores were 0.026 and 0.022, calibration slopes were 1.915 and 1.668, and observed-to-expected ratios were 0.736 and 0.762 in the internal and external cohorts. The preoperative-only RF model achieved ROC-AUCs of 0.884 and 0.905 in the internal and external cohorts, respectively. Decision-curve analysis showed positive RF net benefit across threshold probabilities of 0.01–0.38 internally and 0.01–0.49 externally.

**Conclusion:**

The present machine-learning framework showed favorable discrimination for perioperative SSI risk enrichment after closed pilon fracture surgery, with RF serving as the main high-specificity model. However, limited RF sensitivity and calibration uncertainty suggest that it should be viewed as a preliminary targeted-surveillance tool rather than a rule-out or implementation-ready system. Further recalibration and prospective multicenter validation are needed before routine clinical use.

## Introduction

Pilon fractures are uncommon but disproportionately severe injuries of the distal tibial plafond. Contemporary reviews estimate that they account for approximately 3%–10% of tibial fractures and less than 1% of all lower-extremity fractures, yet their clinical burden is substantial because they are typically high-energy, intra-articular injuries accompanied by metaphyseal comminution and marked soft-tissue compromise (1, 2). Even after modern staged management and advances in fixation, long-term outcomes remain unsatisfactory for many patients, with persistent pain, stiffness, swelling, impaired walking ability, and reduced health-related quality of life (2, 3). Within this already challenging setting, surgical site infection is among the most consequential complications after operative treatment. SSI increases healthcare utilization and costs, prolongs treatment, and may trigger repeated debridement, reconstructive procedures, prolonged antibiotic therapy, chronic osteomyelitis, arthrodesis, or even limb loss in severe cases (4–7). Early identification of patients at increased risk for SSI after pilon fracture surgery therefore remains an important but incompletely solved clinical problem.

Research on SSI prediction after pilon fracture surgery has advanced, but the field remains methodologically constrained. Existing pilon-specific studies have largely relied on conventional regression-based approaches and have identified clinically plausible factors such as soft-tissue injury severity, fracture severity, preoperative delay, operative duration, glycemic status, nutritional status, and surgical approach (8–10). Representative examples include the prospective single-center nomogram by Xie et al. and the retrospective nomogram by Ke et al., both of which helped define the clinical landscape of risk but were still built within relatively narrow modeling frameworks (8, 9). These studies are valuable, yet several gaps remain. Most models were developed in single-center cohorts, relied mainly on static clinical variables or isolated laboratory parameters, and provided limited external validation. More importantly, SSI after pilon fracture is unlikely to arise from a single linear pathway. It more plausibly reflects the interaction of local soft-tissue injury, host inflammatory-metabolic vulnerability, and perioperative treatment complexity. Machine learning offers a potential advantage in this context because it can better accommodate nonlinearity and interaction structure, although broader prediction-model research has also shown that machine learning does not automatically outperform logistic regression and must therefore be paired with rigorous validation and transparent interpretation (11–13). Recent systematic evaluations of SSI prediction models have likewise highlighted that many published machine-learning models remain incompletely validated and methodologically heterogeneous (11, 12). Taken together, these observations suggest that the real need is not simply for a more complex model, but for an externally validated and clinically interpretable framework that integrates routine perioperative variables with systemic inflammatory biomarkers.

Accordingly, the present study aimed to develop and externally validate an explainable machine - learning model for perioperative SSI risk - stratification and early postoperative surveillance support after surgical treatment of closed pilon fractures by integrating conventional clinical predictors with preoperative systemic inflammatory biomarkers.

## Methods

### Study design and patient population

This retrospective multicenter study was conducted to develop and externally validate machine learning models for predicting surgical site infection after operative treatment of closed pilon fractures. Consecutive eligible patients treated at our institution between January 2020 and December 2024 were included in the internal dataset, whereas patients treated at an independent external institution between August 2024 and September 2025 were retrospectively screened using the same eligibility criteria and included in the external validation cohort. Patients were eligible if they were aged 18 years or older, had a confirmed diagnosis of pilon fracture based on the Rüedi and Allgöwer classification, and underwent surgical treatment at the participating institutions. Patients were excluded if they had open or pathological fractures, multiple fractures, preoperative systemic or local infection, a history of autoimmune disease, malignancy, or immunosuppressive therapy, incomplete or missing key baseline, laboratory, or outcome data, or loss to follow-up. Before complete-case eligibility screening, 1,970 internal records and 397 external records were reviewed. Ninety-four internal records and 38 external records were excluded because of incomplete key data, and variable-level missingness in these excluded records is summarized in Supplementary Table S7. All injuries included in the present study were closed pilon fractures, and all patients received standardized perioperative antibiotic prophylaxis according to institutional protocols. For machine learning analysis, the entire internal dataset was randomly divided into a training set and a test set at a ratio of 7:3, while the external cohort was reserved for independent validation. The study was approved by the relevant institutional ethics committees, and the requirement for informed consent was waived owing to the retrospective design and de-identification of patient data. The study was conducted in accordance with the Declaration of Helsinki and the STROCSS guidelines (14). During or after the data collection period, the authors did not have access to any information that could identify individual participants.

### Data collection and candidate predictors

Clinical information was extracted from the electronic medical record system, imaging data were retrieved from the picture archiving and communication system, and laboratory data were obtained from the laboratory information system. Candidate predictors included demographic and injury-related variables, perioperative factors, and laboratory indices. Specifically, the candidate set covered age, body mass index, surgical delay, Rüedi and Allgöwer classification, Tscherne soft-tissue classification coded as grades 0–3, American Society of Anesthesiologists score, surgical approach, intraoperative blood loss, fasting blood glucose, systemic inflammatory response index, Glasgow Prognostic Score, erythrocyte sedimentation rate, high-sensitivity C-reactive protein (hs-CRP), and other clinically relevant perioperative variables.

Fasting blood samples used to derive laboratory biomarkers were collected from peripheral veins on the first morning after admission and before surgery. Reported inflammatory and metabolic indicators, including white blood cells (WBC), neutrophils (NEU), lymphocytes (LYM), serum albumin (ALB), erythrocyte sedimentation rate (ESR), high-sensitivity C-reactive protein (hs-CRP), and fasting blood glucose (FBG), were directly extracted from the routine laboratory database. In addition to these conventional inflammatory and metabolic markers, we further derived a panel of systemic inflammation- and nutrition-related composite biomarkers according to prespecified formulas and definitions, including the systemic immune-inflammation index (SII), calculated as platelet count (×10⁹/L) × neutrophil count (×10⁹/L)/lymphocyte count (×10⁹/L); the systemic inflammation response index (SIRI), calculated as neutrophil count (×10⁹/L) × monocyte count (×10⁹/L)/lymphocyte count (×10⁹/L); the neutrophil-to-lymphocyte ratio (NLR), calculated as neutrophil count (×10⁹/L)/lymphocyte count (×10⁹/L); the platelet-to-lymphocyte ratio (PLR), calculated as platelet count (×10⁹/L)/lymphocyte count (×10⁹/L); the high-sensitivity C-reactive protein-to-lymphocyte ratio (HCLR), calculated as hs-CRP (mg/L)/lymphocyte count (×10⁹/L); the platelet-to-albumin ratio (PAR), calculated as platelet count (×10⁹/L)/albumin (g/L); the prognostic nutritional index (PNI), calculated as albumin (g/L) + 5 × lymphocyte count (×10⁹/L); the Glasgow Prognostic Score (GPS), defined as 0 when hs-CRP was <10 mg/L and albumin was ≥35 g/L, 1 when either hs-CRP was ≥10 mg/L or albumin was <35 g/L, and 2 when hs-CRP was ≥10 mg/L and albumin was <35 g/L; and the C-reactive protein–albumin–lymphocyte index (CALLY), calculated as albumin (g/L) × lymphocyte count (×10⁹/L)/hs-CRP (mg/L).

### Diagnosis of SSI

The diagnostic criteria for SSI were based on the definitions of the Centers for Disease Control and Prevention (15). All inpatient medical records, laboratory pathogen culture results, and imaging studies were comprehensively reviewed. To ensure complete identification of SSI events, all patients were routinely followed up by telephone for more than 12 months postoperatively, which also allowed detection of infections diagnosed or treated at other institutions. For patients who reported SSI during follow-up but lacked corresponding documentation within our hospital system, written confirmation from the treating external institution was requested. For each reported episode, the timing of symptom onset and treatment was reviewed to determine whether it fell within the 30-day (superficial incisional SSI) or 90-day (deep incisional SSI) CDC diagnostic windows; only those events were classified as SSI and included in the primary outcome. Superficial and deep incisional SSI were combined as the primary binary endpoint because both are CDC-defined postoperative SSI events within the same SSI surveillance framework, and deep SSI was too rare in this cohort to support stable subtype-specific model development or validation. We therefore used a combined SSI endpoint to preserve event information. Infections first occurring beyond 90 days were not counted in the main analysis. The determination of SSI was independently performed by two orthopedic specialists, and disagreements were resolved through discussion with a senior chief orthopedic surgeon.

### Statistical analysis

Continuous variables were summarized as mean ± standard deviation or median with interquartile range, as appropriate, whereas categorical variables were summarized as counts and percentages. To reduce dimensionality and identify informative predictors, least absolute shrinkage and selection operator regression was performed in the training set. Predictors with nonzero coefficients at the 1-standard-error rule were retained for subsequent analyses. After feature selection, Spearman correlation analysis was performed among the retained variables to assess pairwise associations and visually inspect potential collinearity before downstream modeling. LASSO regression was implemented with the glmnet framework, and predictors with nonzero coefficients at the one-standard-error rule were retained for downstream modeling (16).

The internal cohort was split into a training set and internal test set at a 7:3 ratio using stratified sampling with random seed. LASSO feature selection was not nested within each cross-validation fold, and this is acknowledged as a possible source of optimism.

A multivariable logistic regression model was constructed using the predictors retained by LASSO regression. This analysis was performed to provide an interpretable statistical comparison with the machine learning models and to identify factors independently associated with postoperative SSI. Odds ratios (ORs) with 95% confidence intervals were calculated, and a two-sided *P* value < 0.05 was considered statistically significant.

Five machine learning algorithms were developed using the predictors selected by LASSO regression, including logistic regression, decision tree, random forest, extreme gradient boosting, and naïve Bayes. Hyperparameters were tuned within the training set by stratified ten-fold cross-validation. The area under the receiver operating characteristic curve was used as the primary discrimination metric during model tuning.

Categorical predictors were one-hot encoded; sparse nominal levels were collapsed using a 1% threshold; novel levels were handled during prediction; and zero-variance predictors were removed. Continuous variables were not manually transformed unless otherwise specified. The final analytical datasets had complete data for the candidate predictors and outcome after eligibility screening. Median/mode imputation steps were retained in the machine-learning recipe only as safeguards during resampling and prediction, and they did not replace the complete-case eligibility screening used to define the primary analytical cohort. Because missingness in the excluded records was concentrated and high for several laboratory and derived inflammatory variables, we did not use multiple imputation to reconstruct the primary analytical cohort; missing-data proportions are reported in Supplementary Table S7. Hyperparameters for decision tree, RF, XGBoost, and naive Bayes were tuned using 30 space-filling grid combinations within stratified ten-fold cross-validation. The selected RF parameters were mtry = 3, trees = 1,103, and min_*n* = 5. The selected XGBoost parameters were mtry = 12, trees = 690, min_*n* = 3, tree_depth = 15, learn_rate = 0.00728, loss_reduction = 9.61e-9, and sample_size = 0.707.

Because SSI was a low-incidence outcome, threshold optimization was additionally performed using out-of-fold predictions from the training-set cross-validation process. For each model, an optimal classification threshold was selected according to a prespecified composite score emphasizing F1 performance while retaining discrimination. The optimized threshold obtained from the cross-validation stage was then fixed and applied unchanged to both the internal test set and the external validation cohort.

No synthetic oversampling, undersampling, or class weighting was applied. The classification threshold was selected using a prespecified score equal to 0.7 x F1 + 0.3 x AUC from out-of-fold predictions and then fixed for internal and external validation.

Model performance was evaluated in the internal test set and in the external validation cohort. Performance metrics included ROC-AUC, PR-AUC, accuracy, sensitivity, specificity, precision/PPV, NPV, F1 score, balanced accuracy, Brier score, calibration intercept, calibration slope, observed-to-expected ratio, and decision-curve analysis. Bootstrap 95% confidence intervals were calculated using 1,000 resamples. Internal-external cohort differences were summarized by standardized mean differences. Receiver operating characteristic curves were generated for all models in the internal test set and the external validation cohort. Confusion matrices were further generated for the best-performing model to illustrate classification performance in different datasets. ROC analyses were performed using standard methods for receiver-operating characteristic analysis (17).

A sensitivity analysis was performed, in which we constructed a preoperative-only RF model without incorporating surgical approach and intraoperative blood loss.

To explain the behavior of the best-performing machine learning model, SHAP analysis was performed for the random forest model. Global feature importance was summarized by mean absolute SHAP values, and a SHAP summary plot was used to visualize the direction and magnitude of feature effects on model output. In addition, permutation importance analysis was conducted for all machine learning models to assess whether important predictors were consistently identified across different algorithms. SHAP analysis was interpreted according to established tree-based explainable AI methods (18).

All statistical analyses were conducted using R software (version 4.5.3; R Foundation for Statistical Computing).

## Results

### Patient characteristics

**Figure 1:**
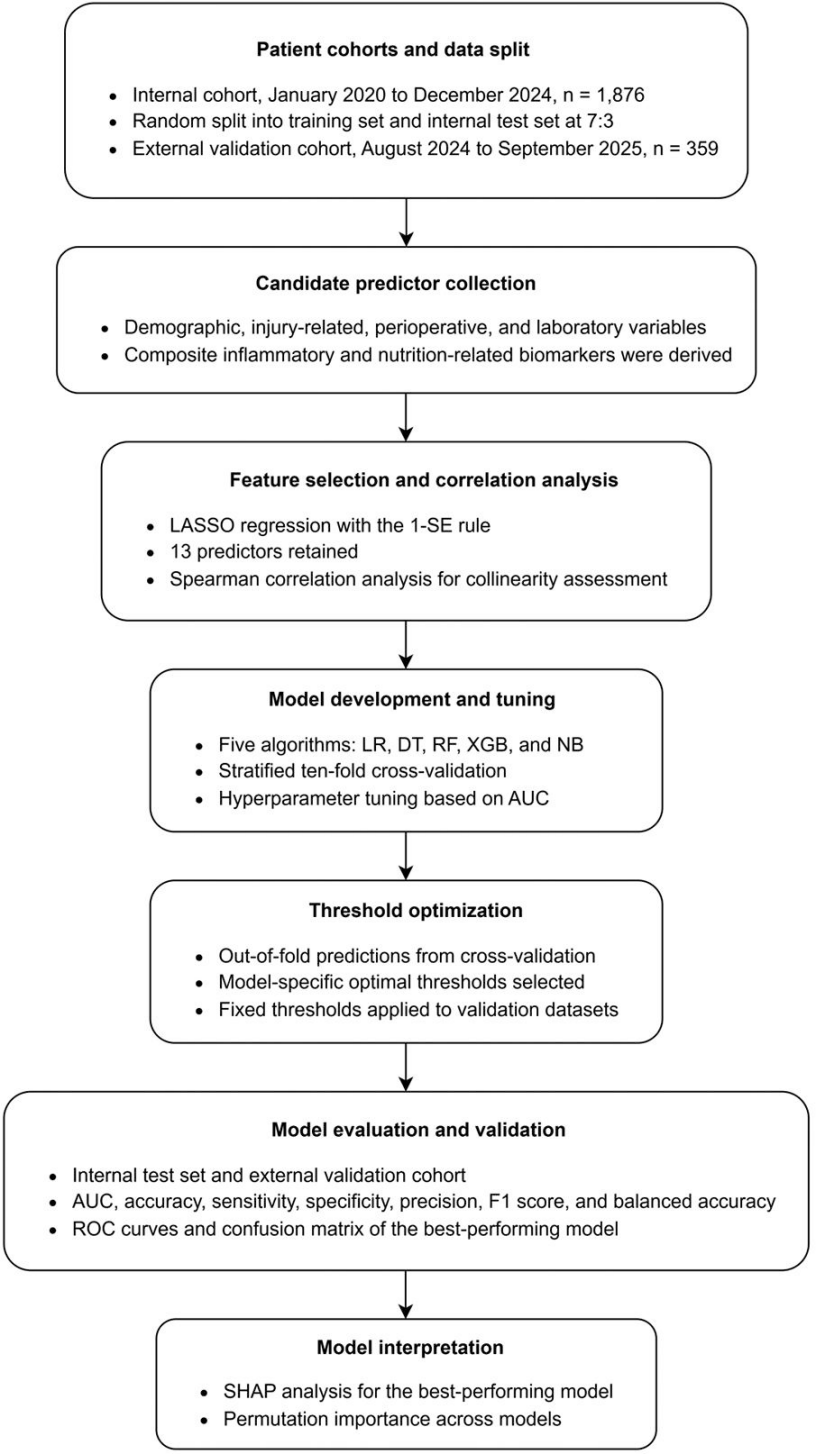
Revised study flowchart of patient screening, dataset partitioning, model development, validation, and added revision analyses.

The study flow is summarized in Figure 1. A total of 1,876 patients were included in the internal dataset, among whom 74 developed SSI, corresponding to an overall event rate of 3.9%. The internal SSI events included 66 superficial incisional SSI events and 8 deep incisional SSI events. After random partitioning, the training set comprised 1,313 patients, including 57 SSI events (51 superficial and 6 deep), and was retained as the main baseline table for model development (Table 1). The internal test set comprised 563 patients, including 17 SSI events (15 superficial and 2 deep), and the independent external validation cohort consisted of 359 patients, including 11 SSI events (10 superficial and 1 deep); their baseline characteristics are provided separately in Supplementary Table S1, and SSI subtype distributions are summarized in Supplementary Supplementary Table S8. In the training set, patients who developed SSI were generally older, had higher BMI, longer surgical delay, more severe fracture and Tscherne soft-tissue classifications, were more likely to undergo a multiple-incision approach, and had higher FBG, ESR, hs-CRP, SIRI, and HCLR levels than those without SSI (Table 1).

**Table 1:** Baseline characteristics of the training cohort •.

Characteristic	Non-SSI group *N* = 1,256	SSI group *N* = 57	*P*
Age	39.00 [27.00, 58.00]	47.00 [33.00, 65.00]	0.004^a^
Sex			0.667
Man	842 (67.0%)	40 (70.2%)	
Woman	414 (33.0%)	17 (29.8%)	
BMI (kg/m²)	25.38 [23.27, 27.30]	27.00 [23.94, 28.00]	0.019^a^
Residence			0.881
Urban	887 (70.6%)	41 (71.9%)	
Rural	369 (29.4%)	16 (28.1%)	
Alcohol use	843 (67.1%)	42 (73.7%)	0.318
Currently smoking	437 (34.8%)	30 (52.6%)	0.009^a^
CCI	1.00 [0.00, 2.00]	1.00 [0.00, 2.00]	0.360
0	357 (28.4%)	16 (28.1%)	0.288
1–2	622 (49.5%)	33 (57.9%)	
≥3	277 (22.1%)	8 (14.0%)	
Preoperative comorbidities			
Hypertension	325 (25.9%)	14 (24.6%)	0.875
Diabetes	99 (7.9%)	6 (10.5%)	0.615
Cerebrovascular disease	64 (5.1%)	3 (5.3%)	1.000
Heart disease	81 (6.4%)	4 (7.0%)	1.000
Chronic respiratory disease	6 (0.5%)	0 (0.0%)	1.000
Liver disease	50 (4.0%)	3 (5.3%)	0.737
Kidney disease	50 (4.0%)	3 (5.3%)	0.737
Malignancy	16 (1.3%)	0 (0.0%)	0.636
Mechanism of injury			0.039^a^
High falling	420 (33.4%)	17 (29.8%)	
Traffic injury	450 (35.8%)	14 (24.6%)	
Fall down	386 (30.7%)	26 (45.6%)	
Surgical delay (days)			0.001^a^
<2	745 (59.3%)	22 (38.6%)	
2–5	255 (20.3%)	12 (21.1%)	
≥6	256 (20.4%)	23 (40.4%)	
Rüedi and Allgöwer classification			0.021^a^
I	191 (15.2%)	7 (12.3%)	
II	527 (42.0%)	15 (26.3%)	
III	538 (42.8%)	35 (61.4%)	
Tscherne classification			0.018^a^
Grade 0	213 (17.0%)	4 (7.0%)	
Grade 1	572 (45.5%)	21 (36.8%)	
Grade 2	315 (25.1%)	19 (33.3%)	
Grade 3	156 (12.4%)	13 (22.8%)	
ASA score			0.116
I	130 (10.4%)	6 (10.5%)	
II	896 (71.3%)	33 (57.9%)	
III	228 (18.2%)	18 (31.6%)	
IV	2 (0.2%)	0 (0.0%)	
Anesthesia method			0.443
General	906 (72.1%)	44 (77.2%)	
Regional	350 (27.9%)	13 (22.8%)	
Surgical duration (minutes)	114.00 [72.00, 151.00]	109.00 [80.00, 155.00]	0.578
Intraoperative blood loss (mL)	131.00 [83.00, 191.00]	155.00 [112.00, 197.00]	0.036^a^
Surgical fixation methods			0.824
ORIF with plate	910 (72.5%)	41 (71.9%)	
ORIF with screws	102 (8.1%)	6 (10.5%)	
CRIF with percutaneous screws	244 (19.4%)	10 (17.5%)	
Surgical approach			0.036^a^
Single incision	918 (73.1%)	34 (59.6%)	
Multiple incisions	338 (26.9%)	23 (40.4%)	
Bone graft			0.152
No	1,137 (90.5%)	48 (84.2%)	
Yes	119 (9.5%)	9 (15.8%)	
Antibiotics type^b^			0.754
1st cephalosporin	1,068 (85.0%)	51 (89.5%)	
2nd cephalosporin	55 (4.4%)	1 (1.8%)	
3rd cephalosporin	78 (6.2%)	3 (5.3%)	
Other antibiotic	55 (4.4%)	2 (3.5%)	
Postoperative antibiotic use (days)	1.00 [1.00, 2.00]	1.00 [1.00, 2.00]	0.254
WBC (*10⁹/L)	10.12 [7.40, 12.52]	9.10 [7.43, 14.93]	0.933
NEU (*10⁹/L)	6.53 [4.03, 7.90]	6.39 [3.64, 9.40]	0.467
LYM (*10⁹/L)	1.98 [1.23, 2.89]	1.83 [1.23, 2.53]	0.569
ALB (g/L)	36.10 [33.00, 39.53]	35.60 [34.10, 40.10]	0.954
ESR (mm/h)	20.54 [12.17, 28.54]	22.40 [16.85, 32.00]	0.029^a^
hs-CRP (mg/L)	5.47 [2.99, 7.94]	6.58 [4.14, 8.41]	0.035^a^
FBG (mmol/L)	5.77 [4.82, 6.64]	6.46 [5.32, 7.51]	<0.001^a^
CONUT	3.00 [2.00, 5.00]	4.00 [2.00, 6.00]	0.130
PNI	42.34 [38.38, 47.11]	40.60 [39.08, 47.10]	0.616
GPS	0.00 [0.00, 1.00]	1.00 [0.00, 1.00]	0.081
SII	567.65 [326.24, 1,009.40]	549.58 [297.38, 1,338.32]	0.746
SIRI	2.36 [1.46, 3.56]	2.73 [2.00, 4.12]	0.048^a^
NLR	2.97 [1.67, 5.56]	3.05 [2.00, 6.80]	0.414
PLR	99.05 [68.28, 150.55]	96.22 [72.34, 165.16]	0.998
HCLR	2.73 [1.45, 4.55]	3.41 [2.21, 5.23]	0.027^a^
PAR	5.24 [4.38, 6.48]	5.05 [3.88, 6.72]	0.556
CALLY	264.74 [121.47, 2,903.92]	149.96 [86.84, 2,036.10]	0.026^a^

a Statistical significance.

b Antibiotics were administered within 30 min before incision and discontinued within 24 h postoperatively, per WHO guidelines. Cefazolin (1st) was the primary choice, with cefuroxime (2nd), ceftriaxone (3rd), or clindamycin (other antibiotic, for recent alcohol use or beta-lactam allergy) used as alternatives. Duration was extended for specific clinical needs based on judgment.

•Values are median [Q1–Q3] for continuous variables and *n* (%) for categorical variables.

SSI, surgical site infection; BMI, body mass index; ASA, American Society of Anesthesiologists; CCI, Charlson comorbidity index; ORIF, open reduction and internal fixation; CRIF, closed reduction and internal fixation; WBC, white blood cell; NEU, neutrophil; LYM, lymphocyte; ALB, albumin; ESR, erythrocyte sedimentation rate; hs-CRP, high-sensitivity C-reactive protein; FBG, fasting blood glucose; CONUT, controlling nutritional status; PNI, prognostic nutritional index; GPS, Glasgow prognostic score; SII, systemic immune inflammation index; SIRI, systemic inflammation response index; NLR, neutrophil-to-lymphocyte ratio; PLR, platelet-to-lymphocyte ratio; HCLR, high-sensitivity C-reactive protein-to-lymphocyte ratio; PAR, platelet-to-albumin ratio; CALLY, C-reactive protein-albumin-lymphocyte index.

Internal-external cohort comparisons showed several case-mix differences. The largest standardized mean differences were observed for WBC (SMD 1.282), lymphocyte count (SMD 0.898), neutrophil count (SMD 0.883), NLR (SMD 0.877), SII (SMD 0.686), CALLY (SMD 0.637), PLR (SMD 0.614), HCLR (SMD 0.451), SIRI (SMD 0.334), sex (SMD 0.328), PLT (SMD 0.227), PAR (SMD 0.213), anesthesia method (SMD 0.171), surgical fixation methods (SMD 0.165), and surgical delay (SMD 0.161).

### Feature selection and correlation analysis

**Figure 2:**
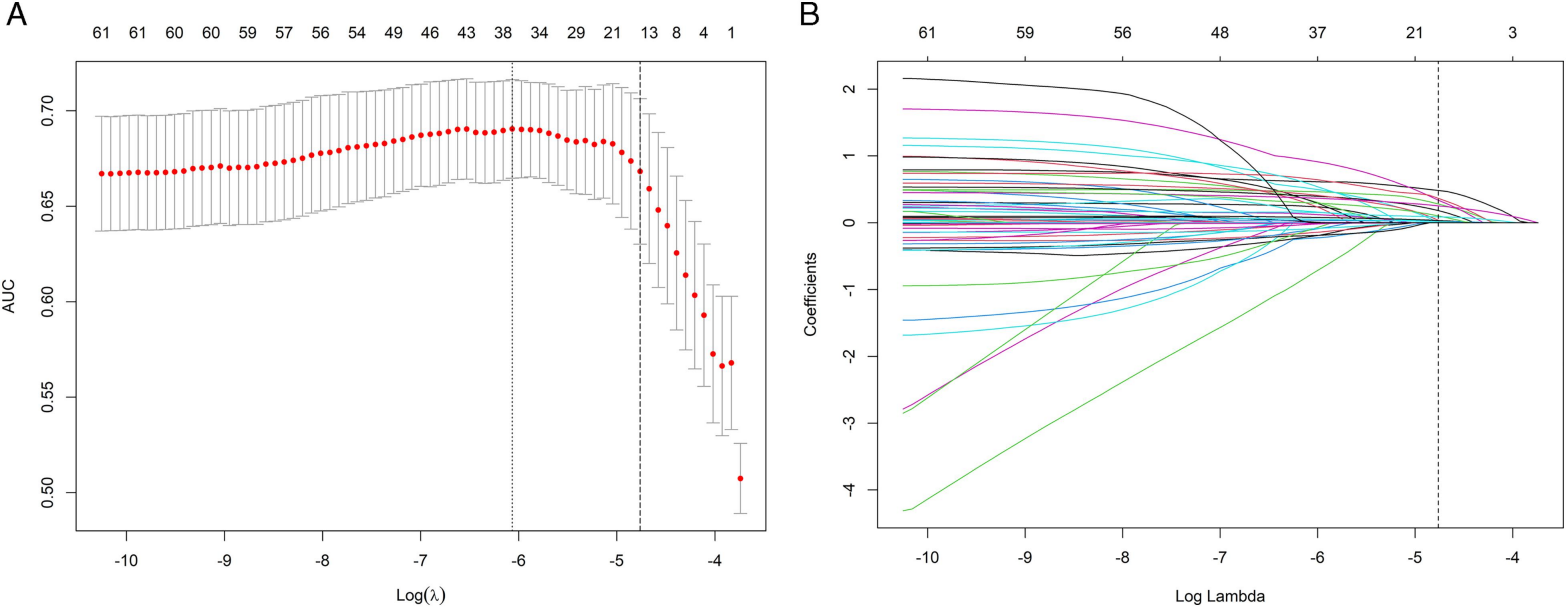
LASSO feature selection. **(A)** Ten-fold cross-validation curve. **(B)** Coefficient trajectories across log(lambda) values.

**Figure 3:**
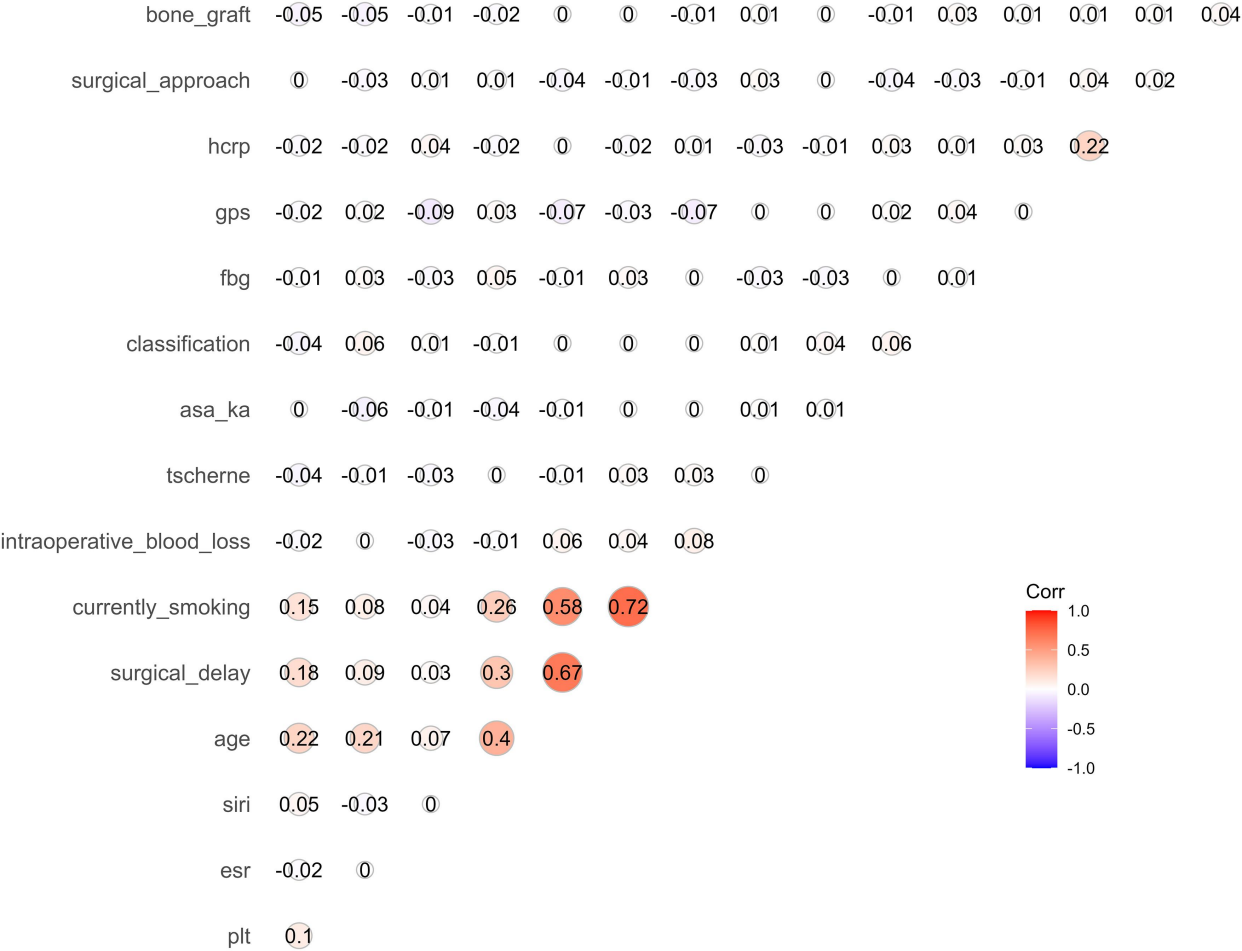
Spearman correlation heat map of the 16 predictors retained by LASSO regression.

LASSO regression retained 16 predictors with nonzero coefficients at the 1-standard-error rule (Figures 2A,B). These predictors were surgical delay, ASA score, Tscherne soft-tissue classification, fracture classification, fasting blood glucose, surgical approach, Glasgow Prognostic Score, systemic inflammatory response index, bone graft, body mass index, erythrocyte sedimentation rate, high-sensitivity C-reactive protein (hs-CRP), current smoking status, age, intraoperative blood loss, and platelet count. These variables were subsequently entered into the correlation analysis, multivariable logistic regression analysis, and machine learning models. The Spearman correlation heat map did not demonstrate pairwise associations of sufficient magnitude to preclude the simultaneous inclusion of these variables in downstream modeling (Figure 3).

### Multivariable logistic regression analysis

In the multivariable logistic regression model based on the 16 LASSO-retained predictors, Tscherne grade 2 (OR 3.366, 95% CI, 1.191–12.087; *P* = 0.035), Tscherne grade 3 (OR 4.950, 95% CI, 1.618–18.605; *P* = 0.009), FBG (OR 1.529, 95% CI, 1.243–1.889; *P* < 0.001), BMI (OR 1.105, 95% CI, 1.004–1.220; *P* = 0.043), ESR (OR 1.043, 95% CI, 1.014–1.073; *P* = 0.004), and PLT (OR 0.991, 95% CI, 0.983–0.999; *P* = 0.029) were associated with SSI. Multiple-incision surgical approach (OR 1.761, 95% CI, 0.954–3.205; *P* = 0.066), SIRI (OR 1.137, 95% CI, 0.984–1.300; *P* = 0.068), and intraoperative blood loss per 100 mL (OR 1.391, 95% CI, 0.969–1.997; *P* = 0.073) showed borderline associations. The full multivariable logistic regression results are shown in Table 2.

**Table 2:** Multivariable logistic regression analysis of factors associated with postoperative SSI.

Variables	*P* value	Odds Ratio	95% CI
Tscherne classification			
Grade 0	1.000		
Grade 1	0.314	1.78	0.63–6.39
Grade 2	0.035^a^	3.37	1.19–12.09
Grade 3	0.009^a^	4.95	1.62–18.61
Surgical delay (days)			
<2	1.000		
2–5	0.851	1.10	0.38–3.07
≥6	0.123	2.28	0.79–6.43
ASA score			
I	1.000		
II	0.366	0.63	0.25–1.88
III/IV	0.313	1.73	0.63–5.40
FBG (mmol/L)	<0.001^a^	1.53	1.24–1.89
Rüedi and Allgöwer classification			
I	1.000		
II	0.541	0.74	0.29–2.05
III	0.280	1.63	0.71–4.24
Surgical approach			
Single incision	1.000		
Multiple incisions	0.066	1.76	0.95–3.21
GPS	0.113	1.49	0.91–2.45
SIRI	0.068	1.14	0.98–1.30
Bone graft			
No	1.000		
Yes	0.149	1.84	0.76–4.07
BMI (kg/m²)	0.043^a^	1.10	1.00–1.22
ESR (mm/h)	0.004^a^	1.04	1.01–1.07
hs-CRP (mg/L)	0.159	1.07	0.97–1.17
Currently smoking			
No	1.000		
Yes	0.745	1.15	0.51–2.66
Age (years)	0.481	1.01	0.98–1.03
Intraoperative blood loss (per 100 mL)	0.073	1.39	0.97–2.00
PLT	0.029^a^	0.99	0.98–1.00

a Statistical significance.

SSI, surgical site infection; OR, odds ratio; CI, confidence interval; ASA, American Society of Anesthesiologists; BMI, body mass index; FBG, fasting blood glucose; ESR, erythrocyte sedimentation rate; hs-CRP, high-sensitivity C-reactive protein; SIRI, systemic inflammation response index; GPS, Glasgow prognostic score; PLT, platelet count.

### Cross-validation performance of machine learning models

**Figure 4:**
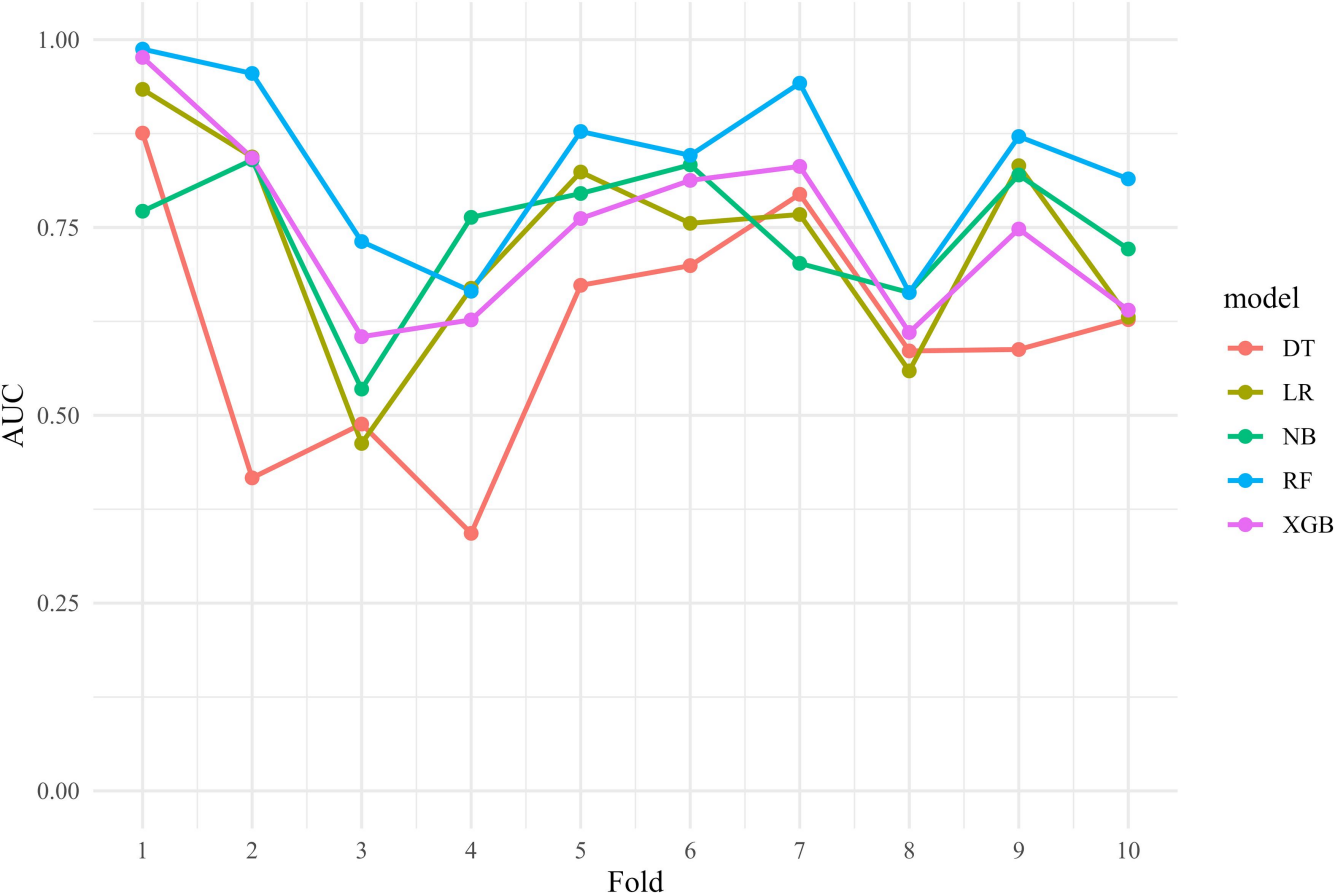
Fold-wise ROC-AUC of five machine-learning models during ten-fold cross-validation in the training set.

Among the five machine learning algorithms, the random forest model achieved the highest mean AUC during ten-fold cross-validation in the training set, followed by XGB, NB, LR, and DT, with mean AUCs of 0.835, 0.745, 0.745, 0.728, and 0.609, respectively (Figure 4). These results indicated that ensemble-based algorithms, particularly RF, provided superior discrimination during model development.

Threshold optimization based on out-of-fold cross-validation predictions yielded model-specific optimal thresholds of 0.12 for LR, 0.09 for DT, 0.17 for RF, 0.10 for XGB, and 0.01 for NB. These optimized thresholds were fixed for all subsequent validation analyses. For descriptive reference, the final RF model in the training set correctly classified 1,252 non-SSI cases and all 57 SSI cases, with only 4 false-positive classifications and no missed SSI events (Figure 6B).

### Performance in the internal test set

In the internal test set, RF achieved ROC-AUC 0.899 (95% CI, 0.831–0.953), PR-AUC 0.297 (95% CI, 0.107–0.544), sensitivity 0.294 (95% CI, 0.077–0.533), specificity 0.987 (95% CI, 0.976–0.996), PPV 0.417 (95% CI, 0.125–0.722), NPV 0.978 (95% CI, 0.965–0.989), F1 score 0.345 (95% CI, 0.109–0.552), balanced accuracy 0.641 (95% CI, 0.533–0.759), and Brier score 0.026 (95% CI, 0.016–0.037) (Supplementary Table S2).

**Figure 5:**
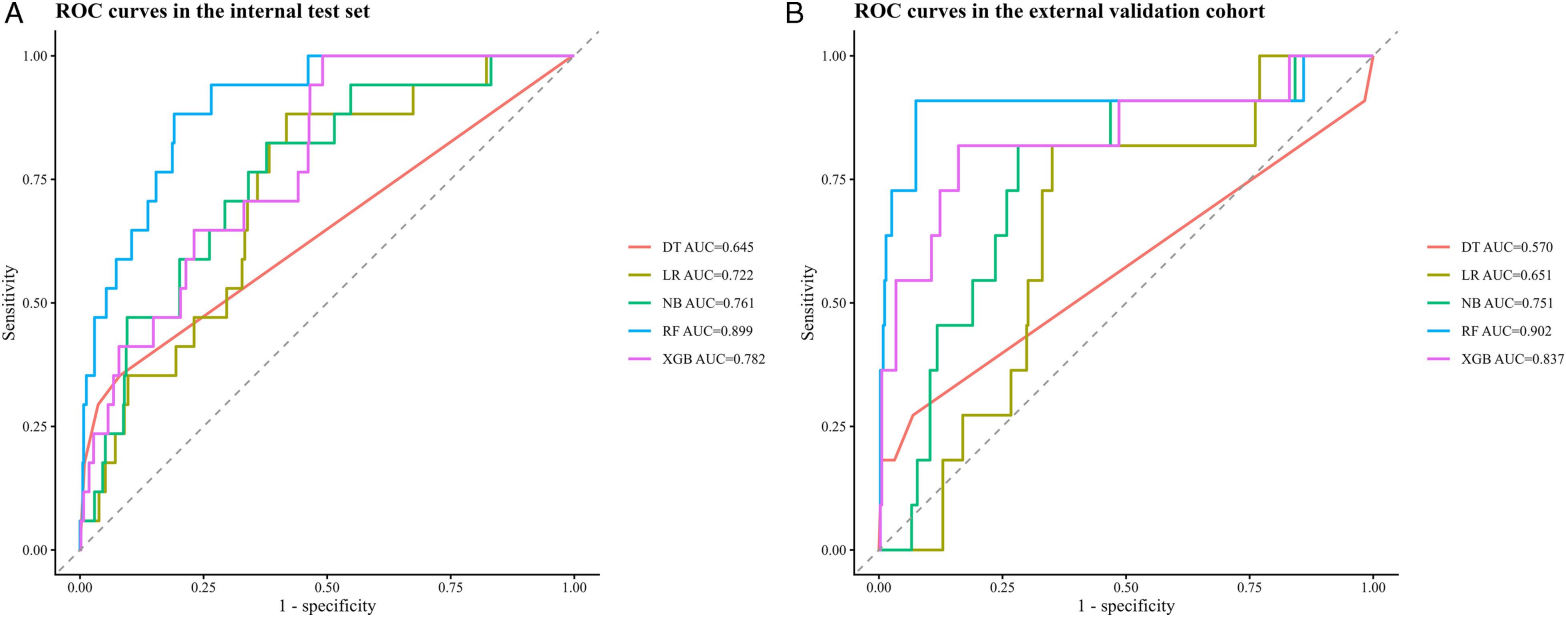
ROC curves of five machine-learning models in **(A)** the internal test set and **(B)** the external validation cohort.

**Figure 6:**
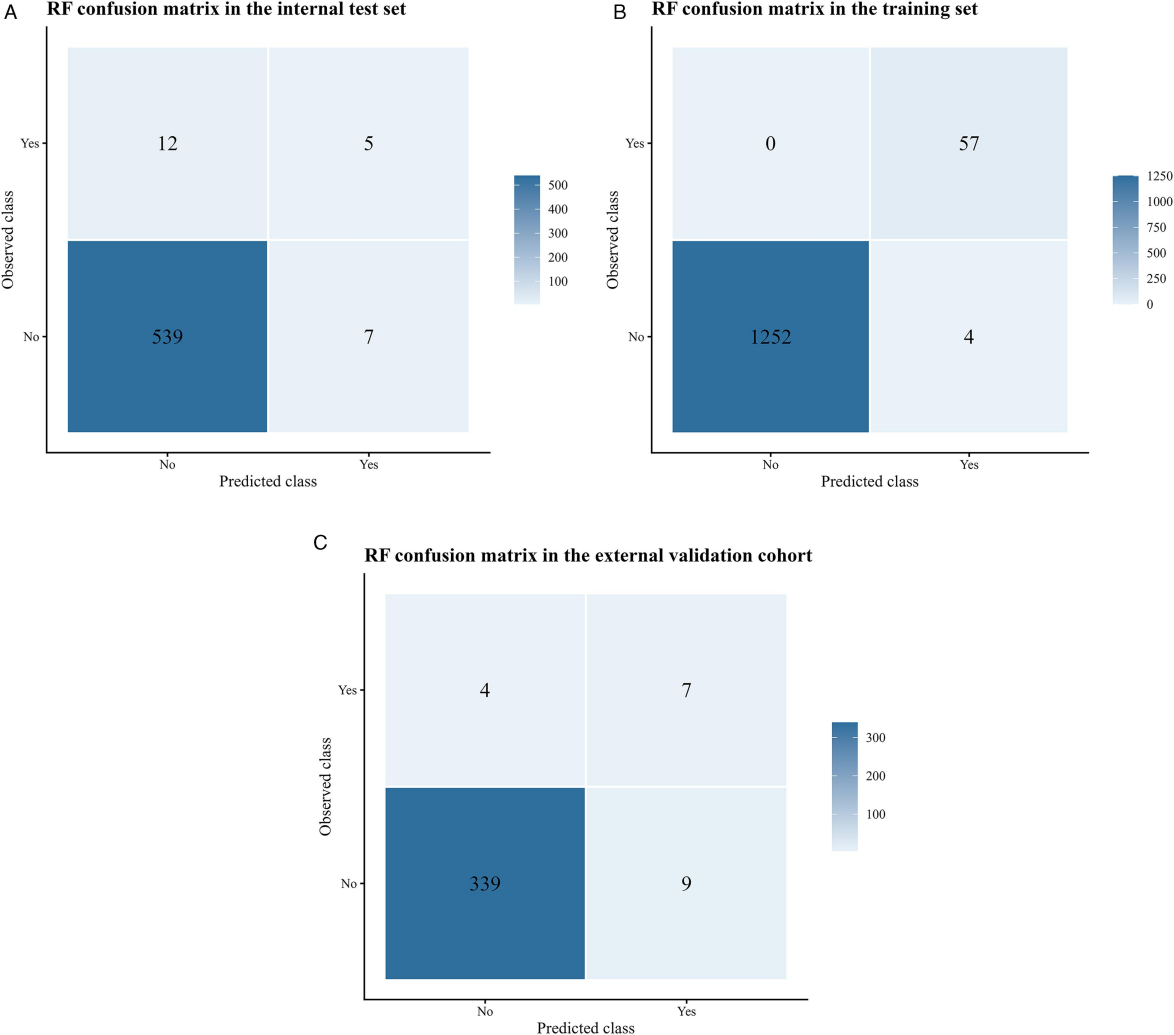
Confusion matrices of the RF model in **(A)** the internal test set, **(B)** the training set, and **(C)** the external validation cohort.

The receiver operating characteristic curves for all five models in the internal test set are shown in Figure 5A. The confusion matrix of the RF model in the internal test set is shown in Figure 6A. Specifically, 539 non-SSI cases and 5 SSI cases were correctly classified, whereas 7 non-SSI cases were misclassified as SSI and 12 SSI cases were missed. These findings indicated that the RF model preserved high specificity in the internal test set but had limited sensitivity for positive event detection.

### Performance in the external validation cohort

In the external validation cohort, RF achieved ROC-AUC 0.902 (95% CI, 0.707–0.991), PR-AUC 0.460 (95% CI, 0.196–0.835), sensitivity 0.636 (95% CI, 0.333–0.909), specificity 0.974 (95% CI, 0.957–0.989), PPV 0.438 (95% CI, 0.182–0.700), NPV 0.988 (95% CI, 0.976–0.997), F1 score 0.519 (95% CI, 0.240–0.733), balanced accuracy 0.805 (95% CI, 0.647–0.942), and Brier score 0.022 (95% CI, 0.012–0.034) (Supplementary Table S2).

RF correctly identified 5 of 17 SSI cases in the internal test set and 7 of 11 SSI cases in the external cohort. XGBoost showed lower sensitivity than RF in the external cohort (0.545 vs. 0.636) and lower specificity and precision in both validation datasets. These findings support interpreting RF as a high-specificity risk-enrichment model rather than a rule-out model.

### Calibration and decision-curve analysis

Calibration assessment showed modest overprediction of absolute risk. For RF, calibration intercepts were −0.332 and −0.314, calibration slopes were 1.915 and 1.668, and observed-to-expected ratios were 0.736 and 0.762 in the internal test and external validation cohorts, respectively (Supplementary Table S3 and Supplementary Figure S1).

Decision-curve analysis showed positive RF net benefit across threshold probabilities of 0.01–0.38 in the internal test set and 0.01–0.49 in the external validation cohort. Net benefit exceeded the treat-all strategy across thresholds from 0.01 to 0.50 internally and from 0.02 to 0.50 externally (Supplementary Table S5).

### Sensitivity analyses

The preoperative-only RF model excluded surgical approach and intraoperative blood loss and used 14 predictors. It achieved ROC-AUC 0.884, PR-AUC 0.282, sensitivity 0.235, specificity 0.976, PPV 0.235, NPV 0.976, F1 score 0.235, balanced accuracy 0.606, and Brier score 0.026 in the internal test set. In the external validation cohort, it achieved ROC-AUC 0.905, PR-AUC 0.410, sensitivity 0.636, specificity 0.960, PPV 0.333, NPV 0.988, F1 score 0.437, balanced accuracy 0.798, and Brier score 0.023 (Supplementary Table S4).

### SHAP analysis of the random forest model

**Figure 7:**
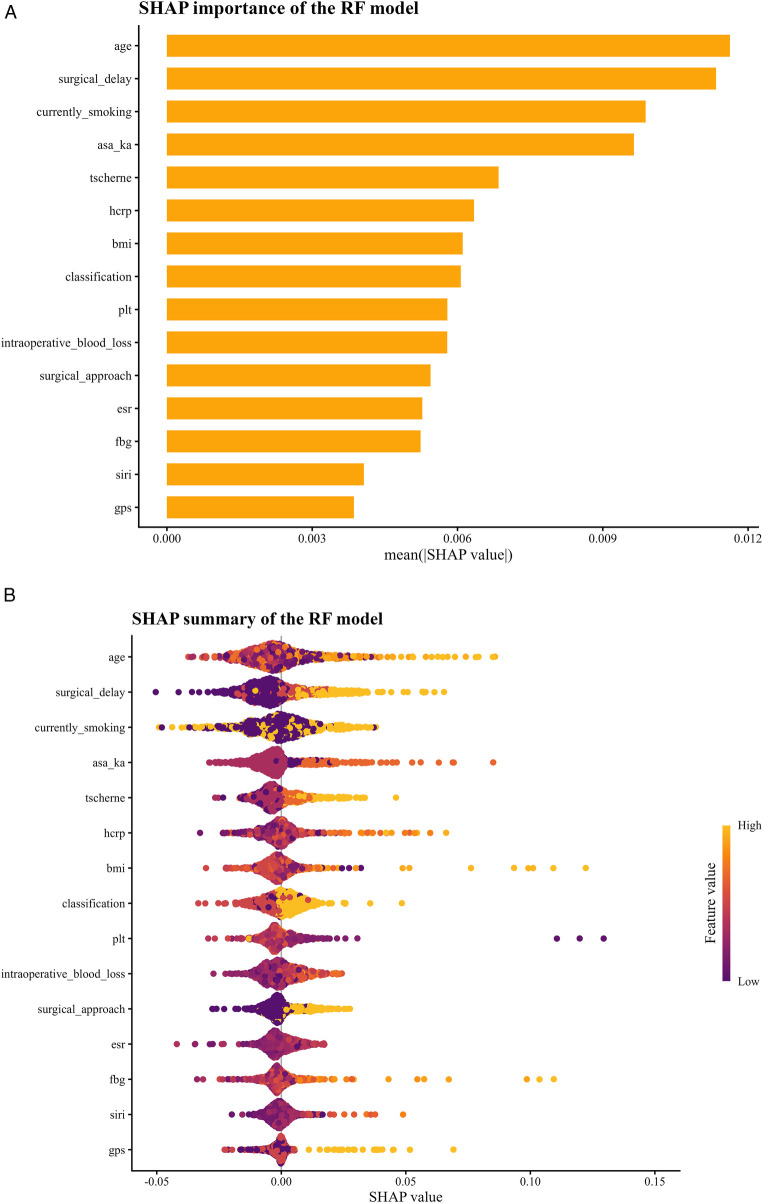
RF explainability analysis. **(A)** Mean absolute SHAP importance ranking. **(B)** SHAP summary plot. These analyses indicate contributions to model output and should not be interpreted causally.

Because RF exhibited the best overall performance across the validation datasets, SHAP analysis was performed for the RF model. The mean absolute SHAP values demonstrated that age exerted the greatest contribution to model output, followed by surgical delay, current smoking status, ASA score, Tscherne classification, hs-CRP, BMI, fracture classification, platelet count, and intraoperative blood loss (Figure 7A). The SHAP summary plot further suggested that older age, longer surgical delay, more severe soft-tissue injury, higher ASA grade, current smoking, and a greater inflammatory burden generally pushed the model toward a higher predicted probability of SSI (Figure 7B).

SHAP and permutation-importance analyses were interpreted as model-explanation tools rather than causal analyses. These variables should not be interpreted as necessarily causing SSI; for example, surgical delay may reflect soft-tissue severity, injury complexity, staged management, or institutional practice, whereas surgical approach and blood loss may partly reflect treatment complexity.

### Permutation importance analysis

**Figure 8:**
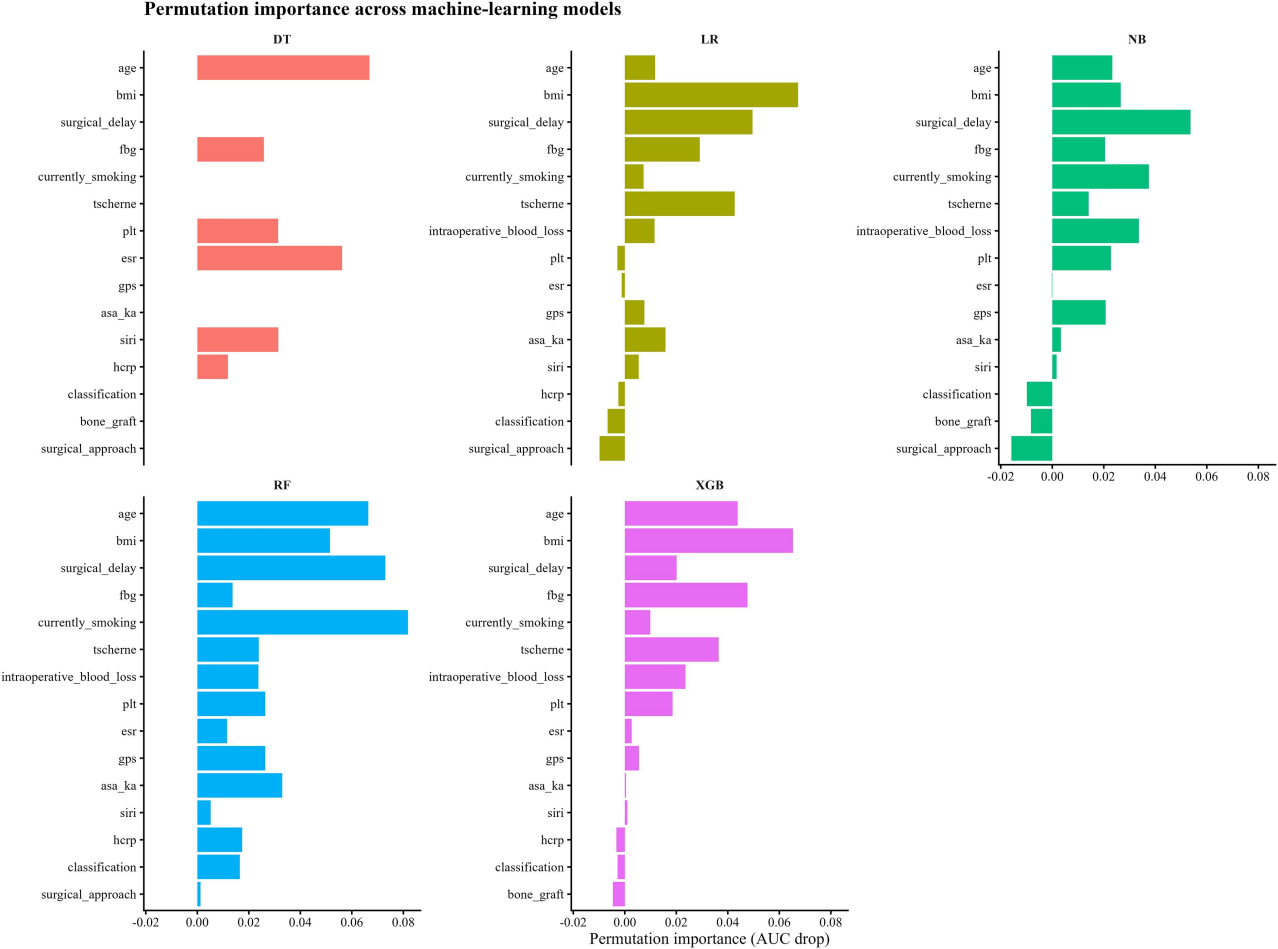
Permutation importance of predictors across five machine-learning models, expressed as the decrease in ROC-AUC after feature shuffling.

Permutation importance analysis yielded a broadly consistent feature ranking across the different machine learning models. In the RF model, the most important predictors included current smoking status, surgical delay, age, BMI, ASA score, Glasgow Prognostic Score, platelet count, Tscherne classification, intraoperative blood loss, and hs-CRP. In the XGB model, BMI, fasting blood glucose, age, Tscherne classification, intraoperative blood loss, surgical delay, and platelet count were ranked highly. Across all algorithms, several variables repeatedly appeared among the most influential predictors, particularly surgical delay, Tscherne classification, BMI, age, fasting blood glucose, platelet count, and intraoperative blood loss. These findings suggested that the major predictive signals were not driven by a single algorithm but were relatively stable across different modeling strategies (Figure 8).

## Discussion

In this multicenter retrospective study, we developed and externally validated machine-learning models for predicting postoperative surgical site infection after operative treatment of closed pilon fractures. Within the internal cohort of 1,876 patients, 74 developed SSI, and the external cohort of 359 patients contributed an additional 11 SSI events. LASSO retained 16 predictors spanning soft-tissue status, perioperative timing, operative factors, metabolic indices, inflammatory markers, smoking status, and platelet count. Among the five algorithms, random forest achieved the best overall performance, showing the highest mean AUC during cross-validation and the strongest discrimination in both the internal test set and the external validation cohort. In parallel, the multivariable logistic model identified a more parsimonious but biologically coherent signal, highlighting Tscherne classification, fasting blood glucose, BMI, ESR, and PLT as the major independent correlates of SSI. Taken together, these findings indicate that postoperative SSI after closed pilon fracture does not arise from a single isolated factor, but rather from the combined influence of local soft-tissue injury, host inflammatory-metabolic status, perioperative treatment complexity, and systemic hematologic status.

The present results place particular emphasis on the interplay between local tissue injury and operative timing. Patients who developed SSI tended to have more severe Tscherne soft-tissue injury and longer surgical delay, and this pattern persisted across several analytic layers. In the logistic model, Tscherne grade 2 and grade 3 were independently associated with increased odds of SSI, whereas surgical delay itself was not independently significant after adjustment. By contrast, surgical delay ranked prominently in the explainability analyses, indicating that it remained an important model-level signal even when its adjusted main effect was attenuated. This pattern is clinically plausible. Closed pilon fractures are frequently accompanied by substantial swelling, contusion, and soft-tissue compromise, all of which directly influence wound-healing capacity and local resistance to infection. At the same time, operative delay likely reflects more than time alone. In daily practice, it often represents the combined consequences of soft-tissue condition, fracture complexity, staging strategy, and overall perioperative burden. This interpretation is consistent with the long-standing principles of staged pilon fracture management, which were introduced to reduce wound complications by deferring definitive fixation until the soft-tissue envelope became more favorable (1, 2, 10). It is also supported by recent pilon-specific prediction studies, in which Tscherne classification, fracture severity, preoperative blood sugar, and operative time emerged as key predictors of postoperative SSI (8, 9). Viewed in this context, soft-tissue insult and perioperative timing are best understood as a coupled clinical axis rather than two separate predictors.

This local-injury signal was accompanied by a consistent inflammatory-metabolic and hematologic signature. Fasting blood glucose, BMI, ESR, and PLT remained independently associated with SSI in the multivariable analysis, while hs-CRP, age, smoking status, and intraoperative blood loss also ranked highly in SHAP and permutation analyses. The baseline comparisons pointed in the same direction, as patients with SSI tended to have less favorable glucose and inflammatory profiles. This suggests that the models were not simply recapitulating fracture severity or operative complexity. Instead, they also captured a host-response dimension that may shape susceptibility to infection after injury. Elevated fasting glucose may reflect chronic dysmetabolism in some patients, but in the acute peri-traumatic setting it may also index stress hyperglycemia and impaired systemic control. This interpretation is supported by orthopaedic trauma literature showing that perioperative hyperglycemia, including stress hyperglycemia in patients without known diabetes, is associated with postoperative SSI and deep SSI (19–21). On the inflammatory side, CRP and ESR remain among the most widely used serum markers in orthopaedic infection assessment (22), and recent work in high-risk lower-extremity fractures has likewise suggested that preoperative CRP, ESR, and blood-cell-derived inflammatory indices may carry diagnostic or prognostic value in fracture-related infection contexts (23). These findings are also consistent with a systematic review and meta-analysis of OTA/AO type C tibial plateau and tibial plafond fractures, which highlighted the clinical relevance of SSI risk in these high-energy injuries (24). The inverse association between PLT and SSI in the logistic model should be interpreted cautiously, but its retention by LASSO and recurrent appearance in model-explanation outputs suggest that platelet-related inflammatory or physiologic reserve signals may contribute to risk stratification. Equally noteworthy is that several clinically intuitive variables, such as ASA score, hs-CRP, smoking status, and blood loss, were not always retained as stable independent predictors in the logistic model, yet repeatedly emerged as influential features in the explainability analyses. This pattern suggests that part of the infection signal may be expressed through nonlinearity, interaction, or context dependence rather than through a simple main effect. It also helps explain why the combination of multivariable regression and machine learning is particularly useful in this setting: the former supports interpretability, whereas the latter more fully reflects the composite risk structure underlying SSI after pilon fracture surgery.

The analyses show that the RF model provides favorable discrimination and high specificity, with improved sensitivity in the external validation cohort after the 16-variable LASSO refit, but internal sensitivity remained limited. The model should therefore be considered a preliminary risk-enrichment and targeted-surveillance tool rather than a definitive clinical decision system. Patients flagged as high risk may warrant closer wound surveillance, careful perioperative optimization, and more intensive follow-up, but patients classified as low risk should not be assumed to have negligible SSI risk. The comparison between RF and XGBoost illustrates a clinically important trade-off. RF provided stronger discrimination and specificity, whereas XGBoost offered lower external sensitivity and lower specificity and precision. Model selection and threshold choice should therefore depend on the clinical context, the burden of false positives, and the priority assigned to sensitivity vs. specificity. External validation is a strength of this study, but transportability remains uncertain. The external cohort included only 11 SSI events and differed from the internal cohort in several inflammatory and demographic variables. The observed-to-expected ratio also suggested modest overprediction, indicating that recalibration may be needed before deployment in other centers.

This study has several limitations. First, the retrospective design leaves room for residual confounding and selection bias. Second, the number of SSI events was low, especially in the external validation cohort, limiting precision and increasing the risk of optimism. Third, LASSO feature selection was not nested within each cross-validation fold. Fourth, some predictors included in the final model, such as intraoperative blood loss and surgical approach, are not available at the time of initial preoperative risk assessment. This may limit the use of the full model for early risk stratification before surgery. To address this issue, we developed an additional sensitivity model using only preoperatively available variables. Fifth, 94 internal records and 38 external records were excluded because of incomplete key data before construction of the analytical datasets. Missingness among these excluded records was concentrated in laboratory and derived inflammatory variables, with several variables missing in more than 70% of excluded internal records and more than 80% of excluded external records. We therefore reported the missing-data structure rather than using multiple imputation to reconstruct the primary cohort; nevertheless, complete-case eligibility screening may introduce selection bias and may limit generalizability. Sixth, superficial and deep SSI were analyzed as a combined CDC-defined SSI endpoint to preserve event information for this rare outcome. This approach improved statistical stability but may obscure subtype-specific predictors, particularly because deep SSI occurred in only 8 internal patients and 1 external patient. Finally, SHAP and permutation importance explain model output but do not establish causality.

## Conclusion

The present machine-learning framework, with RF as the primary high-specificity model, provided favorable discrimination for perioperative SSI risk enrichment after closed pilon fracture surgery. However, RF sensitivity was limited, and absolute risk estimates require cautious interpretation. Therefore, the framework should be viewed as a preliminary tool for targeted surveillance rather than as a rule-out instrument or a ready-to-implement clinical decision system. Further recalibration, prospective multicenter validation, and clinical utility assessment are needed before routine implementation.

## Data Availability

The datasets presented in this article are not readily available because of privacy and ethical restrictions related to patient confidentiality. Data are available from the Institutional Data Access/Ethics Committee of Hebei Medical University Third Hospital (contact via Ethics Committee of Hebei Medical University Third Hospital at ydsyllwyh@hebmu.edu.cn) for researchers who meet the criteria for access to confidential data.
