# Supplementary material for: Explainable machine learning for perioperative surgical site infection risk enrichment after operative treatment of closed pilon fractures: a multicenter retrospective study with external validation
Source: Front Surg. 2026 Jul 6;13:1850385. doi: 10.3389/fsurg.2026.1850385 (PMC13381018; doi:10.3389/fsurg.2026.1850385)

Supplementary Table S1. Baseline characteristics of the internal test and external validation cohorts

| Characteristic | Internal test set | | | External validation cohort | | |
| --- | --- | --- | --- | --- | --- | --- |
|  | Non-SSI group N = 546 | SSI group N = 17 | P | Non-SSI group N = 348 | SSI group N = 11 | P |
| Age | 40.00 [26.00, 59.00] | 55.00 [34.00, 65.00] | 0.036* | 39.00 [27.00, 58.00] | 45.00 [40.50, 68.50] | 0.014* |
| Sex |  |  | 0.217 |  |  | 0.767 |
| Man | 334 (61.2%) | 13 (76.5%) |  | 171 (49.1%) | 6 (54.5%) |  |
| Woman | 212 (38.8%) | 4 (23.5%) |  | 177 (50.9%) | 5 (45.5%) |  |
| BMI (kg/m²) | 25.20 [22.84, 26.99] | 27.50 [26.68, 29.26] | <0.001* | 25.40 [23.40, 27.00] | 23.90 [21.81, 27.50] | 0.565 |
| Residence |  |  | 0.282 |  |  | 0.505 |
| Urban | 395 (72.3%) | 10 (58.8%) |  | 257 (73.9%) | 7 (63.6%) |  |
| Rural | 151 (27.7%) | 7 (41.2%) |  | 91 (26.1%) | 4 (36.4%) |  |
| Alcohol use | 353 (64.7%) | 17 (100.0%) | 0.003* | 231 (66.4%) | 8 (72.7%) | 0.762 |
| Currently smoking | 188 (34.4%) | 9 (52.9%) | 0.118 | 129 (37.1%) | 7 (63.6%) | 0.100 |
| CCI | 1.00 [0.25, 2.00] | 2.00 [1.00, 3.00] | 0.198 | 1.00 [0.75, 2.00] | 1.00 [0.50, 2.00] | 0.271 |
| 0 | 137 (25.1%) | 3 (17.6%) | 0.556 | 87 (25.0%) | 3 (27.3%) | 0.162 |
| 1–2 | 277 (50.7%) | 8 (47.1%) |  | 174 (50.0%) | 8 (72.7%) |  |
| ≥3 | 132 (24.2%) | 6 (35.3%) |  | 87 (25.0%) | 0 (0.0%) |  |
| **Preoperative comorbidities** |  |  |  |  |  |  |
| Hypertension | 151 (27.7%) | 3 (17.6%) | 0.435 | 98 (28.2%) | 1 (9.1%) | 0.200 |
| Diabetes | 51 (9.3%) | 4 (23.5%) | 0.077 | 29 (8.3%) | 0 (0.0%) | 0.608 |
| Cerebrovascular disease | 26 (4.8%) | 2 (11.8%) | 0.208 | 19 (5.5%) | 0 (0.0%) | 0.659 |
| Heart disease | 50 (9.2%) | 3 (17.6%) | 0.389 | 30 (8.6%) | 0 (0.0%) | 0.607 |
| Chronic respiratory disease | 2 (0.4%) | 0 (0.0%) | 1.000 | 1 (0.3%) | 0 (0.0%) | 1.000 |
| Liver disease | 16 (2.9%) | 1 (5.9%) | 1.000 | 14 (4.0%) | 1 (9.1%) | 0.378 |
| Kidney disease | 20 (3.7%) | 1 (5.9%) | 1.000 | 17 (4.9%) | 0 (0.0%) | 0.679 |
| Malignancy | 8 (1.5%) | 1 (5.9%) | 0.244 | 6 (1.7%) | 0 (0.0%) | 1.000 |
| Mechanism of injury |  |  | 0.006* |  |  | <0.001* |
| High falling | 197 (36.1%) | 1 (5.9%) |  | 126 (36.2%) | 1 (9.1%) |  |
| Traffic injury | 181 (33.2%) | 5 (29.4%) |  | 127 (36.5%) | 1 (9.1%) |  |
| Fall down | 168 (30.8%) | 11 (64.7%) |  | 95 (27.3%) | 9 (81.8%) |  |
| Surgical delay (days) |  |  | 0.002* |  |  | 0.791 |
| <2 | 318 (58.2%) | 3 (17.6%) |  | 182 (52.3%) | 7 (63.6%) |  |
| 2–5 | 126 (23.1%) | 7 (41.2%) |  | 69 (19.8%) | 2 (18.2%) |  |
| ≥6 | 102 (18.7%) | 7 (41.2%) |  | 97 (27.9%) | 2 (18.2%) |  |
| Rüedi and Allgöwer classification |  |  | 0.887 |  |  | 0.578 |
| I | 72 (13.2%) | 3 (17.6%) |  | 37 (10.6%) | 2 (18.2%) |  |
| II | 207 (37.9%) | 6 (35.3%) |  | 141 (40.5%) | 3 (27.3%) |  |
| III | 267 (48.9%) | 8 (47.1%) |  | 170 (48.9%) | 6 (54.5%) |  |
| Tscherne classification |  |  | 0.305 |  |  | 0.889 |
| Grade 0 | 85 (15.6%) | 2 (11.8%) |  | 56 (16.1%) | 1 (9.1%) |  |
| Grade 1 | 249 (45.6%) | 5 (29.4%) |  | 146 (42.0%) | 6 (54.5%) |  |
| Grade 2 | 131 (24.0%) | 5 (29.4%) |  | 80 (23.0%) | 2 (18.2%) |  |
| Grade 3 | 81 (14.8%) | 5 (29.4%) |  | 66 (19.0%) | 2 (18.2%) |  |
| ASA score |  |  | 1.000 |  |  |  |
| I | 54 (9.9%) | 2 (11.8%) |  | 38 (10.9%) | 2 (18.2%) |  |
| II | 375 (68.7%) | 11 (64.7%) |  | 239 (68.7%) | 4 (36.4%) |  |
| III | 115 (21.1%) | 4 (23.5%) |  | 71 (20.4%) | 5 (45.5%) |  |
| IV | 2 (0.4%) | 0 (0.0%) |  | 0 (0.0%) | 0 (0.0%) |  |
| Anesthesia method |  |  | 0.080 |  |  | 0.508 |
| General | 403 (73.8%) | 16 (94.1%) |  | 278 (79.9%) | 10 (90.9%) |  |
| Regional | 143 (26.2%) | 1 (5.9%) |  | 70 (20.1%) | 1 (9.1%) |  |
| Surgical duration (minutes) | 116.00 [76.25, 153.00] | 137.00 [100.00, 155.00] | 0.102 | 117.50 [77.75, 158.25] | 54.00 [40.00, 127.00] | 0.060 |
| Intraoperative blood loss (mL) | 130.00 [83.00, 180.00] | 134.00 [82.00, 206.00] | 0.541 | 130.00 [82.00, 182.00] | 154.00 [87.00, 170.50] | 0.704 |
| Surgical fixation methods |  |  | 0.840 |  |  | 0.599 |
| ORIF with plate | 400 (73.3%) | 13 (76.5%) |  | 259 (74.4%) | 7 (63.6%) |  |
| ORIF with screws | 50 (9.2%) | 2 (11.8%) |  | 15 (4.3%) | 1 (9.1%) |  |
| CRIF with percutaneous screws | 96 (17.6%) | 2 (11.8%) |  | 74 (21.3%) | 3 (27.3%) |  |
| Surgical approach |  |  | 0.163 |  |  | 0.305 |
| Single incision | 388 (71.1%) | 15 (88.2%) |  | 254 (73.0%) | 10 (90.9%) |  |
| Multiple incisions | 158 (28.9%) | 2 (11.8%) |  | 94 (27.0%) | 1 (9.1%) |  |
| Bone graft |  |  | 0.378 |  |  | 1.000 |
| No | 497 (91.0%) | 17 (100.0%) |  | 318 (91.4%) | 10 (90.9%) |  |
| Yes | 49 (9.0%) | 0 (0.0%) |  | 30 (8.6%) | 1 (9.1%) |  |
| Antibiotics type° |  |  | 0.767 |  |  | 0.357 |
| 1st cephalosporin | 467 (85.5%) | 16 (94.1%) |  | 296 (85.1%) | 10 (90.9%) |  |
| 2nd cephalosporin | 21 (3.8%) | 0 (0.0%) |  | 18 (5.2%) | 0 (0.0%) |  |
| 3rd cephalosporin | 29 (5.3%) | 0 (0.0%) |  | 24 (6.9%) | 0 (0.0%) |  |
| Other antibiotic | 29 (5.3%) | 1 (5.9%) |  | 10 (2.9%) | 1 (9.1%) |  |
| Postoperative antibiotic use (days) | 1.00 [1.00, 2.00] | 1.00 [1.00, 3.00] | 0.652 | 1.00 [1.00, 2.00] | 1.00 [1.00, 1.00] | 0.099 |
| WBC (*10⁹/L) | 10.07 [7.31, 12.52] | 10.59 [8.75, 11.50] | 0.735 | 14.71 [11.89, 16.80] | 16.90 [16.35, 18.48] | <0.001* |
| NEU (*10⁹/L) | 6.38 [3.88, 7.98] | 6.68 [4.47, 7.81] | 0.657 | 7.80 [7.00, 11.00] | 8.40 [7.55, 15.10] | 0.112 |
| LYM (*10⁹/L) | 1.79 [1.23, 2.86] | 2.62 [1.67, 3.27] | 0.113 | 1.23 [0.90, 1.62] | 1.33 [0.98, 1.46] | 0.920 |
| ALB (g/L) | 35.50 [32.90, 39.12] | 34.20 [29.40, 38.44] | 0.134 | 36.12 [33.00, 39.89] | 37.78 [35.70, 39.60] | 0.436 |
| ESR (mm/h) | 21.50 [12.25, 30.20] | 23.53 [8.27, 27.88] | 0.827 | 21.05 [15.19, 26.39] | 24.27 [22.21, 24.67] | 0.154 |
| hs-CRP (mg/L) | 5.37 [2.84, 7.99] | 4.78 [2.04, 7.34] | 0.728 | 5.62 [2.98, 7.99] | 4.64 [3.28, 7.21] | 0.678 |
| FBG (mmol/L) | 5.84 [4.82, 6.61] | 6.65 [5.24, 7.15] | 0.204 | 5.82 [4.86, 6.63] | 6.95 [6.34, 8.22] | 0.008* |
| CONUT | 4.00 [2.00, 5.00] | 4.00 [2.00, 6.00] | 0.335 | 3.00 [2.00, 5.00] | 2.00 [2.00, 5.00] | 0.826 |
| PNI | 41.91 [38.39, 46.50] | 40.30 [36.14, 42.69] | 0.087 | 42.78 [38.14, 47.38] | 44.43 [40.19, 48.35] | 0.445 |
| GPS | 1.00 [0.00, 1.00] | 1.00 [0.00, 1.00] | 0.350 | 0.00 [0.00, 1.00] | 0.00 [0.00, 1.00] | 0.418 |
| SII | 600.12 [342.24, 1,070.70] | 597.55 [385.89, 765.31] | 0.587 | 1,312.54 [825.83, 1,906.09] | 1,767.07 [1,126.69, 2,499.01] | 0.254 |
| SIRI | 2.43 [1.51, 3.61] | 3.14 [2.11, 3.82] | 0.204 | 3.25 [2.26, 3.98] | 2.28 [1.60, 3.73] | 0.113 |
| NLR | 3.07 [1.78, 5.73] | 3.05 [1.85, 3.81] | 0.463 | 7.41 [4.90, 10.15] | 10.38 [5.27, 11.97] | 0.377 |
| PLR | 104.82 [68.93, 157.63] | 95.23 [67.35, 117.80] | 0.150 | 149.99 [108.21, 209.78] | 176.40 [115.75, 214.40] | 0.740 |
| HCLR | 2.69 [1.42, 4.77] | 2.16 [0.96, 3.73] | 0.309 | 4.15 [2.09, 7.18] | 3.97 [2.20, 5.77] | 0.815 |
| PAR | 5.26 [4.47, 6.43] | 6.04 [5.15, 6.98] | 0.189 | 4.91 [4.27, 5.86] | 5.43 [4.93, 6.72] | 0.262 |
| CALLY | 265.08 [119.11, 3,244.00] | 175.49 [87.95, 490.29] | 0.182 | 4,480.49 [3,102.94, 6,109.88] | 4,914.00 [3,188.60, 5,719.25] | 0.856 |

Note: *Statistical significance.

•Values are median [Q1–Q3] for continuous variables and n (%) for categorical variables.

°Antibiotics were administered within 30 min before incision and discontinued within 24 h postoperatively, per WHO guidelines. Cefazolin (1st) was the primary choice, with cefuroxime (2nd), ceftriaxone (3rd), or clindamycin (other antibiotic, for recent alcohol use or beta-lactam allergy) used as alternatives. Duration was extended for specific clinical needs based on judgment.

Abbreviations: SSI, surgical site infection; BMI, body mass index; ASA, American Society of Anesthesiologists; CCI, Charlson comorbidity index; ORIF, open reduction and internal fixation; CRIF, closed reduction and internal fixation; WBC, white blood cell; NEU, neutrophil; LYM, lymphocyte; ALB, albumin; ESR, erythrocyte sedimentation rate; hs-CRP, high-sensitivity C-reactive protein; FBG, fasting blood glucose; CONUT, controlling nutritional status; PNI, prognostic nutritional index; GPS, Glasgow prognostic score; SII, systemic immune inflammation index; SIRI, systemic inflammation response index; NLR, neutrophil-to-lymphocyte ratio; PLR, platelet-to-lymphocyte ratio; HCLR, high-sensitivity C-reactive protein-to-lymphocyte ratio; PAR, platelet-to-albumin ratio; CALLY, C-reactive protein-albumin-lymphocyte index.

Supplementary Table S2. RF validation performance with bootstrap 95% confidence intervals

| dataset | metric | point_estimate | 95% CI |
| --- | --- | --- | --- |
| Internal_Test | AUC | 0.899 | 0.831-0.953 |
| Internal_Test | PR_AUC | 0.297 | 0.107-0.544 |
| Internal_Test | Sensitivity | 0.294 | 0.077-0.533 |
| Internal_Test | Specificity | 0.987 | 0.976-0.996 |
| Internal_Test | Precision | 0.417 | 0.125-0.722 |
| Internal_Test | NPV | 0.978 | 0.965-0.989 |
| Internal_Test | F1 | 0.345 | 0.109-0.552 |
| Internal_Test | Balanced_Accuracy | 0.641 | 0.533-0.759 |
| Internal_Test | Brier | 0.026 | 0.016-0.037 |
| External_Validation | AUC | 0.902 | 0.707-0.991 |
| External_Validation | PR_AUC | 0.460 | 0.196-0.835 |
| External_Validation | Sensitivity | 0.636 | 0.333-0.909 |
| External_Validation | Specificity | 0.974 | 0.957-0.989 |
| External_Validation | Precision | 0.438 | 0.182-0.700 |
| External_Validation | NPV | 0.988 | 0.976-0.997 |
| External_Validation | F1 | 0.519 | 0.240-0.733 |
| External_Validation | Balanced_Accuracy | 0.805 | 0.647-0.942 |
| External_Validation | Brier | 0.022 | 0.012-0.034 |

Supplementary Table S3. Calibration indices of the RF model

| dataset | model | Calibration_Intercept | Calibration_Slope | Observed_Expected | Mean_Predicted_Risk | Observed_Risk |
| --- | --- | --- | --- | --- | --- | --- |
| External_Validation | RF | -0.314 | 1.668 | 0.762 | 0.040 | 0.031 |
| Internal_Test | RF | -0.332 | 1.915 | 0.736 | 0.041 | 0.030 |

Supplementary Table S4. Preoperative-only RF sensitivity model

| dataset | model | AUC | PR_AUC | Accuracy | Sensitivity | Specificity | Precision | NPV | F1 | Balanced_Accuracy | Brier | TP | FP | TN | FN | Threshold | predictor_count |
| --- | --- | --- | --- | --- | --- | --- | --- | --- | --- | --- | --- | --- | --- | --- | --- | --- | --- |
| External_Validation | RF_preoperative_only | 0.905 | 0.410 | 0.950 | 0.636 | 0.960 | 0.333 | 0.988 | 0.437 | 0.798 | 0.023 | 7 | 14 | 334 | 4 | 0.16 | 14 |
| Internal_Test | RF_preoperative_only | 0.884 | 0.282 | 0.954 | 0.235 | 0.976 | 0.235 | 0.976 | 0.235 | 0.606 | 0.026 | 4 | 13 | 533 | 13 | 0.16 | 14 |

Supplementary Table S5. Summary of decision-curve analysis for the RF model

| dataset | model | positive_threshold_min | positive_threshold_max | better_than_all_min | better_than_all_max |
| --- | --- | --- | --- | --- | --- |
| External_Validation | RF | 0.01 | 0.49 | 0.02 | 0.5 |
| Internal_Test | RF | 0.01 | 0.38 | 0.01 | 0.5 |

Supplementary Table S6. Full validation metrics of all five machine-learning models

| dataset | model | AUC | PR_AUC | Accuracy | Sensitivity | Specificity | Precision | NPV | F1 | Balanced_Accuracy | Brier | TP | FP | TN | FN | Threshold |
| --- | --- | --- | --- | --- | --- | --- | --- | --- | --- | --- | --- | --- | --- | --- | --- | --- |
| External_Validation | DT | 0.570 | 0.195 | 0.944 | 0.182 | 0.968 | 0.154 | 0.974 | 0.167 | 0.575 | 0.030 | 2 | 11 | 337 | 9 | 0.09 |
| External_Validation | LR | 0.651 | 0.042 | 0.858 | 0.000 | 0.885 | 0.000 | 0.966 |  | 0.443 | 0.036 | 0 | 40 | 308 | 11 | 0.12 |
| External_Validation | NB | 0.751 | 0.066 | 0.969 | 0.000 | 1.000 |  | 0.969 |  | 0.500 | 0.031 | 0 | 0 | 348 | 11 | 0.01 |
| External_Validation | RF | 0.902 | 0.460 | 0.964 | 0.636 | 0.974 | 0.438 | 0.988 | 0.519 | 0.805 | 0.022 | 7 | 9 | 339 | 4 | 0.17 |
| External_Validation | XGB | 0.837 | 0.269 | 0.925 | 0.545 | 0.937 | 0.214 | 0.985 | 0.308 | 0.741 | 0.026 | 6 | 22 | 326 | 5 | 0.10 |
| Internal_Test | DT | 0.645 | 0.213 | 0.943 | 0.294 | 0.963 | 0.200 | 0.978 | 0.238 | 0.629 | 0.033 | 5 | 20 | 526 | 12 | 0.09 |
| Internal_Test | LR | 0.722 | 0.117 | 0.897 | 0.235 | 0.918 | 0.082 | 0.975 | 0.121 | 0.576 | 0.033 | 4 | 45 | 501 | 13 | 0.12 |
| Internal_Test | NB | 0.761 | 0.089 | 0.970 | 0.000 | 1.000 |  | 0.970 |  | 0.500 | 0.030 | 0 | 0 | 546 | 17 | 0.01 |
| Internal_Test | RF | 0.899 | 0.297 | 0.966 | 0.294 | 0.987 | 0.417 | 0.978 | 0.345 | 0.641 | 0.026 | 5 | 7 | 539 | 12 | 0.17 |
| Internal_Test | XGB | 0.782 | 0.113 | 0.931 | 0.235 | 0.952 | 0.133 | 0.976 | 0.170 | 0.594 | 0.028 | 4 | 26 | 520 | 13 | 0.10 |

Supplementary Table S7. Missing-data proportions in excluded records and screened records

| Dataset | Variable | Missing among excluded records, n (%) | Missing among screened records, n (%) |
| --- | --- | --- | --- |
| Internal/training missing-data file | ALB | 67 (71.3%) | 67 (3.4%) |
| Internal/training missing-data file | CALLY | 67 (71.3%) | 67 (3.4%) |
| Internal/training missing-data file | CONUT | 67 (71.3%) | 67 (3.4%) |
| Internal/training missing-data file | ESR | 67 (71.3%) | 67 (3.4%) |
| Internal/training missing-data file | FBG | 67 (71.3%) | 67 (3.4%) |
| Internal/training missing-data file | GPS | 67 (71.3%) | 67 (3.4%) |
| Internal/training missing-data file | HCLR | 67 (71.3%) | 67 (3.4%) |
| Internal/training missing-data file | hs-CRP | 67 (71.3%) | 67 (3.4%) |
| Internal/training missing-data file | LYM | 67 (71.3%) | 67 (3.4%) |
| Internal/training missing-data file | MON | 67 (71.3%) | 67 (3.4%) |
| Internal/training missing-data file | NEU | 67 (71.3%) | 67 (3.4%) |
| Internal/training missing-data file | NLR | 67 (71.3%) | 67 (3.4%) |
| Internal/training missing-data file | PAR | 67 (71.3%) | 67 (3.4%) |
| Internal/training missing-data file | PLR | 67 (71.3%) | 67 (3.4%) |
| Internal/training missing-data file | PLT | 67 (71.3%) | 67 (3.4%) |
| Internal/training missing-data file | PNI | 67 (71.3%) | 67 (3.4%) |
| Internal/training missing-data file | SII | 67 (71.3%) | 67 (3.4%) |
| Internal/training missing-data file | SIRI | 67 (71.3%) | 67 (3.4%) |
| Internal/training missing-data file | TC | 67 (71.3%) | 67 (3.4%) |
| Internal/training missing-data file | WBC | 67 (71.3%) | 67 (3.4%) |
| Internal/training missing-data file | BMI | 66 (70.2%) | 66 (3.4%) |
| Internal/training missing-data file | Alcohol use | 59 (62.8%) | 59 (3.0%) |
| Internal/training missing-data file | CCI | 59 (62.8%) | 59 (3.0%) |
| Internal/training missing-data file | CCI category | 59 (62.8%) | 59 (3.0%) |
| Internal/training missing-data file | Cerebrovascular disease | 59 (62.8%) | 59 (3.0%) |
| Internal/training missing-data file | Chronic respiratory disease | 59 (62.8%) | 59 (3.0%) |
| Internal/training missing-data file | Currently smoking | 59 (62.8%) | 59 (3.0%) |
| Internal/training missing-data file | Diabetes | 59 (62.8%) | 59 (3.0%) |
| Internal/training missing-data file | Heart disease | 59 (62.8%) | 59 (3.0%) |
| Internal/training missing-data file | Hypertension | 59 (62.8%) | 59 (3.0%) |
| Internal/training missing-data file | Kidney disease | 59 (62.8%) | 59 (3.0%) |
| Internal/training missing-data file | Liver disease | 59 (62.8%) | 59 (3.0%) |
| Internal/training missing-data file | Malignancy | 59 (62.8%) | 59 (3.0%) |
| Internal/training missing-data file | Mechanism of injury | 15 (16.0%) | 15 (0.8%) |
| Internal/training missing-data file | Surgical delay | 15 (16.0%) | 15 (0.8%) |
| External-validation missing-data file | ALB | 31 (81.6%) | 31 (7.8%) |
| External-validation missing-data file | CALLY | 31 (81.6%) | 31 (7.8%) |
| External-validation missing-data file | CONUT | 31 (81.6%) | 31 (7.8%) |
| External-validation missing-data file | ESR | 31 (81.6%) | 31 (7.8%) |
| External-validation missing-data file | FBG | 31 (81.6%) | 31 (7.8%) |
| External-validation missing-data file | GPS | 31 (81.6%) | 31 (7.8%) |
| External-validation missing-data file | HCLR | 31 (81.6%) | 31 (7.8%) |
| External-validation missing-data file | hs-CRP | 31 (81.6%) | 31 (7.8%) |
| External-validation missing-data file | LYM | 31 (81.6%) | 31 (7.8%) |
| External-validation missing-data file | MON | 31 (81.6%) | 31 (7.8%) |
| External-validation missing-data file | NEU | 31 (81.6%) | 31 (7.8%) |
| External-validation missing-data file | NLR | 31 (81.6%) | 31 (7.8%) |
| External-validation missing-data file | PAR | 31 (81.6%) | 31 (7.8%) |
| External-validation missing-data file | PLR | 31 (81.6%) | 31 (7.8%) |
| External-validation missing-data file | PLT | 31 (81.6%) | 31 (7.8%) |
| External-validation missing-data file | PNI | 31 (81.6%) | 31 (7.8%) |
| External-validation missing-data file | SII | 31 (81.6%) | 31 (7.8%) |
| External-validation missing-data file | SIRI | 31 (81.6%) | 31 (7.8%) |
| External-validation missing-data file | TC | 31 (81.6%) | 31 (7.8%) |
| External-validation missing-data file | WBC | 31 (81.6%) | 31 (7.8%) |
| External-validation missing-data file | Alcohol use | 29 (76.3%) | 29 (7.3%) |
| External-validation missing-data file | BMI | 29 (76.3%) | 29 (7.3%) |
| External-validation missing-data file | CCI | 29 (76.3%) | 29 (7.3%) |
| External-validation missing-data file | CCI category | 29 (76.3%) | 29 (7.3%) |
| External-validation missing-data file | Cerebrovascular disease | 29 (76.3%) | 29 (7.3%) |
| External-validation missing-data file | Chronic respiratory disease | 29 (76.3%) | 29 (7.3%) |
| External-validation missing-data file | Currently smoking | 29 (76.3%) | 29 (7.3%) |
| External-validation missing-data file | Diabetes | 29 (76.3%) | 29 (7.3%) |
| External-validation missing-data file | Heart disease | 29 (76.3%) | 29 (7.3%) |
| External-validation missing-data file | Hypertension | 29 (76.3%) | 29 (7.3%) |
| External-validation missing-data file | Kidney disease | 29 (76.3%) | 29 (7.3%) |
| External-validation missing-data file | Liver disease | 29 (76.3%) | 29 (7.3%) |
| External-validation missing-data file | Malignancy | 29 (76.3%) | 29 (7.3%) |
| External-validation missing-data file | Residence | 13 (34.2%) | 13 (3.3%) |
| External-validation missing-data file | Surgical delay | 8 (21.1%) | 8 (2.0%) |
| External-validation missing-data file | Intraoperative blood loss | 6 (15.8%) | 6 (1.5%) |
| External-validation missing-data file | Mechanism of injury | 5 (13.2%) | 5 (1.3%) |
| External-validation missing-data file | Postoperative antibiotic use | 4 (10.5%) | 4 (1.0%) |
| External-validation missing-data file | Surgical duration | 4 (10.5%) | 4 (1.0%) |
| External-validation missing-data file | Antibiotics type | 3 (7.9%) | 3 (0.8%) |
| External-validation missing-data file | Rüedi and Allgöwer classification | 2 (5.3%) | 2 (0.5%) |

Supplementary Table S8. Distribution of superficial and deep surgical site infection events

| Dataset | Total N | All SSI, n (%) | Superficial SSI, n (%) | Deep SSI, n (%) |
| --- | --- | --- | --- | --- |
| Training cohort | 1,313 | 57 (4.3%) | 51 (3.9%) | 6 (0.5%) |
| Internal test cohort | 563 | 17 (3.0%) | 15 (2.7%) | 2 (0.4%) |
| External validation cohort | 359 | 11 (3.1%) | 10 (2.8%) | 1 (0.3%) |

Note: SSI subtypes were adjudicated using the CDC diagnostic framework. The primary model used any superficial or deep SSI as the combined binary endpoint.

Supplementary Figure S1. Calibration curves of the RF model.


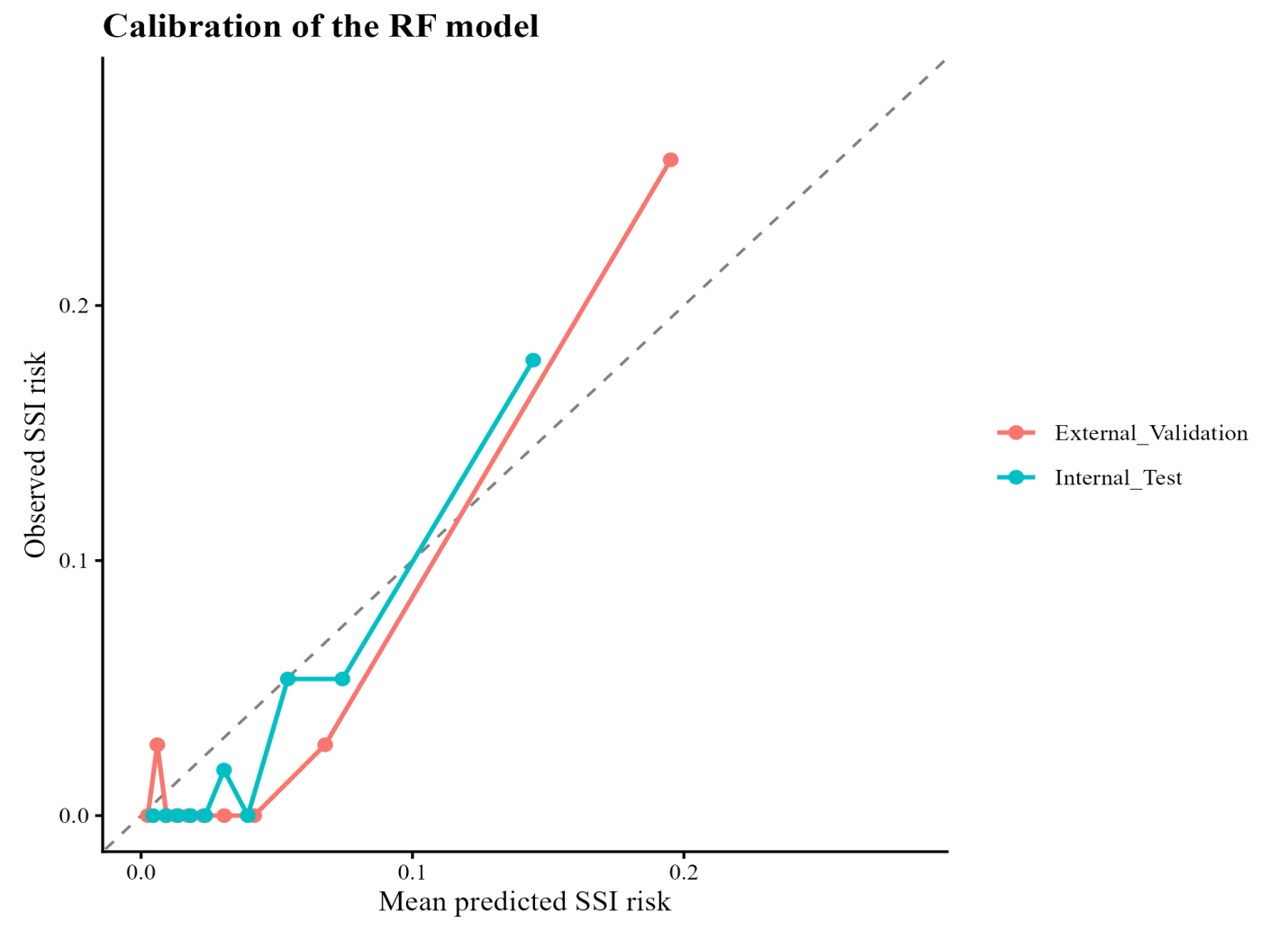


Supplementary Figure S2. Precision-recall curves of the RF model.


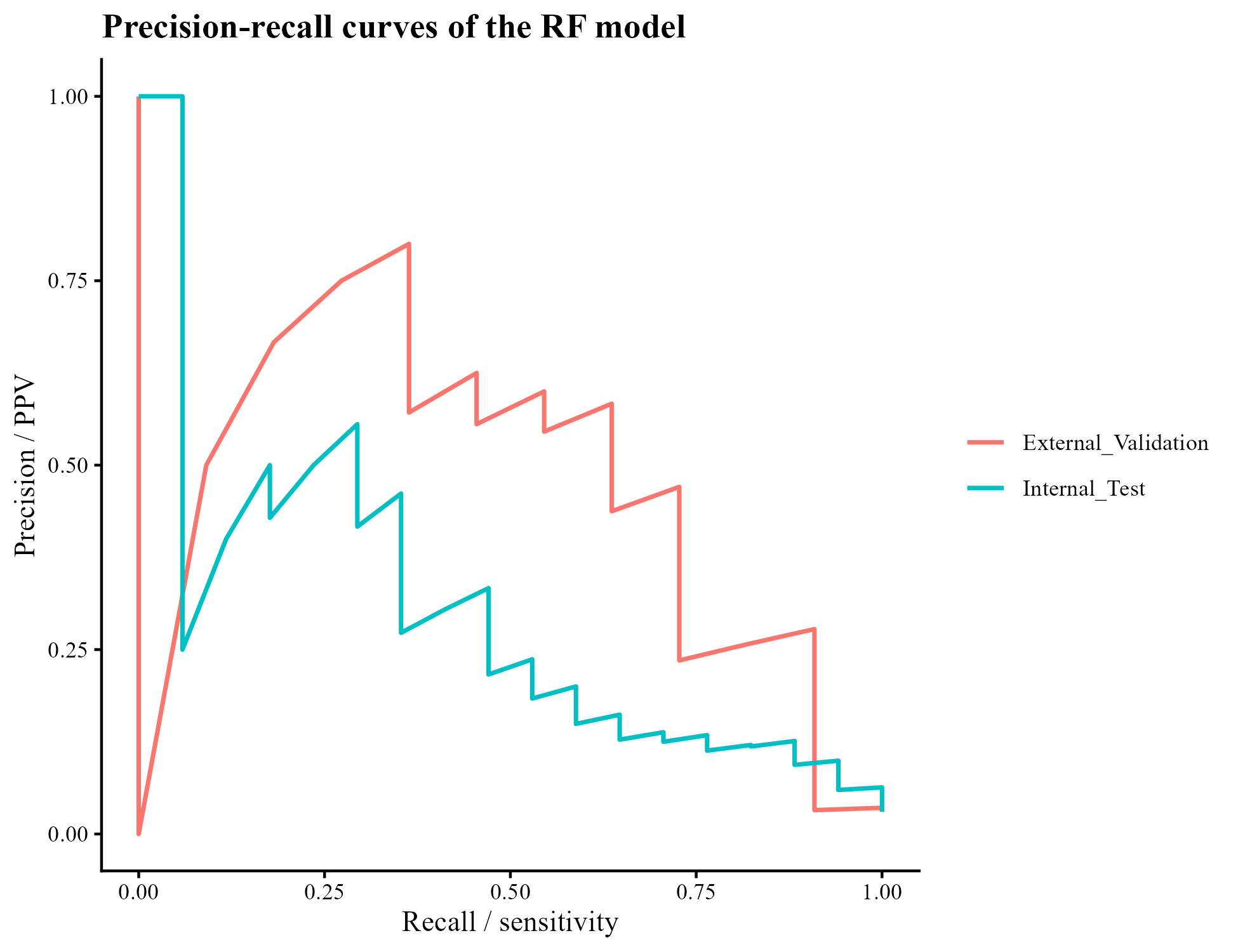


Supplementary Figure S3. Decision-curve analysis of the RF model.


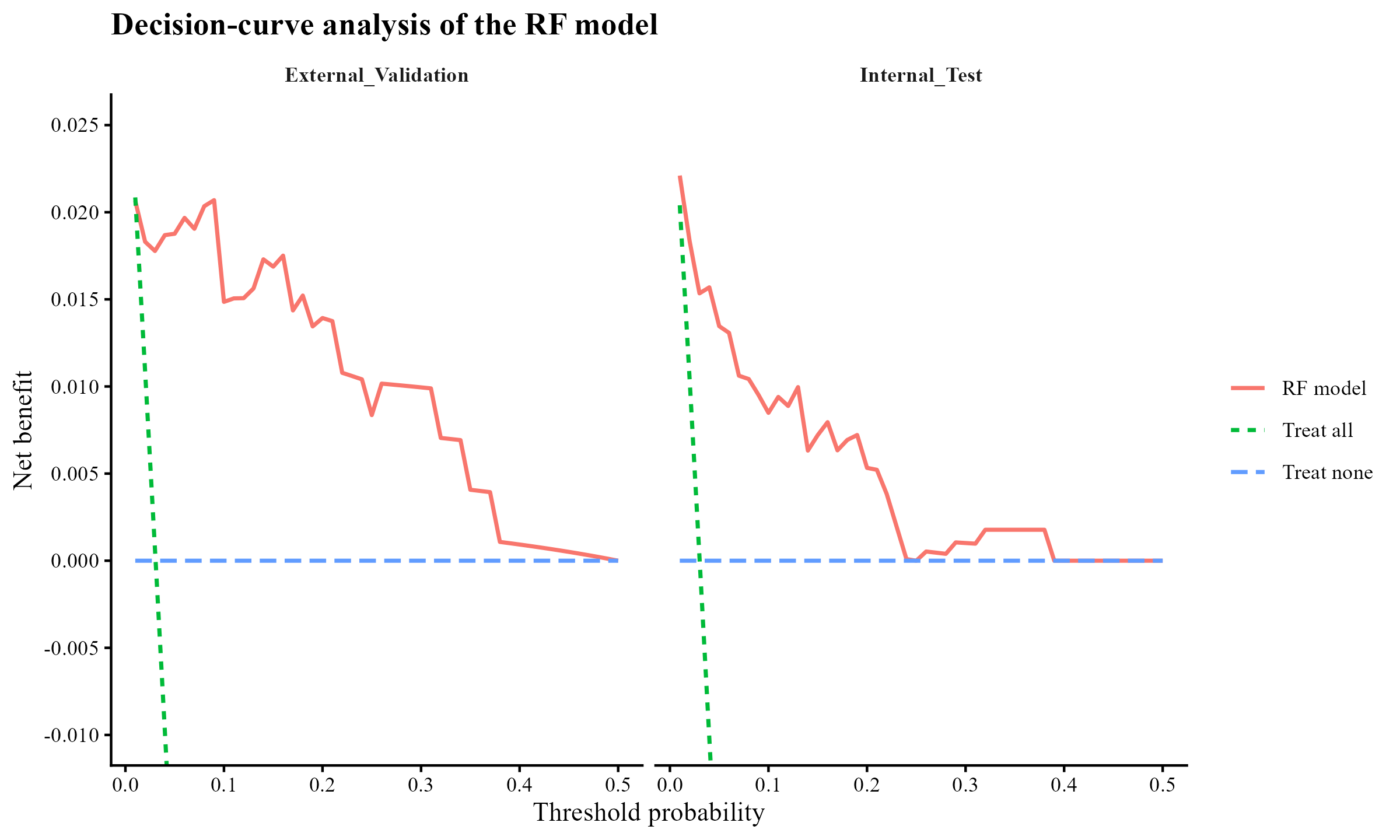


Supplementary Figure S4. Largest standardized mean differences between the internal and external cohorts.


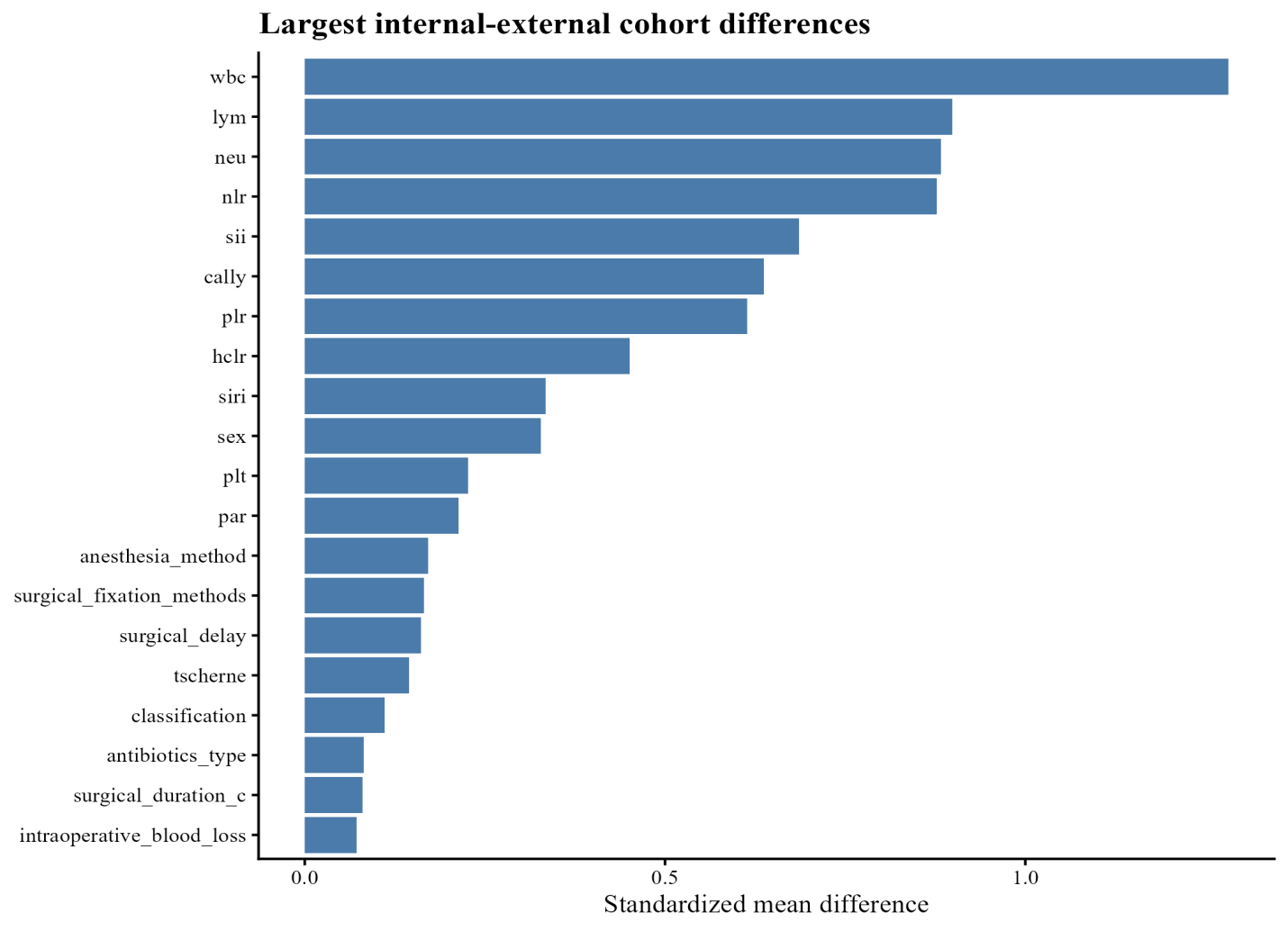

Supplement: Supplementary file 1 [file Supplementaryfile1.docx]
